# *Tead4* and *Tfap2c* generate bipotency and a bistable switch in totipotent embryos to promote robust lineage diversification

**DOI:** 10.1038/s41594-024-01311-9

**Published:** 2024-05-24

**Authors:** Meng Zhu, Maciej Meglicki, Adiyant Lamba, Peizhe Wang, Christophe Royer, Karen Turner, Muhammad Abdullah Jauhar, Celine Jones, Tim Child, Kevin Coward, Jie Na, Magdalena Zernicka-Goetz

**Affiliations:** 1 Mammalian Embryo and Stem Cell Group, Department of Physiology, Development and Neuroscience, University of Cambridge, Cambridge, UK; 2 Division of Biology and Biological Engineering, California Institute of Technology, Pasadena, CA USA; 3 Centre for Stem Cell Biology and Regenerative Medicine, School of Medicine, Tsinghua University, Beijing, China; 4 Department of Physiology, Anatomy and Genetics, University of Oxford, Oxford, UK; 5 Oxford Fertility, Institute of Reproductive Sciences, Oxford, UK; 6 Nuffield Department of Women’s and Reproductive Health, Level 3, Women’s Centre, John Radcliffe Hospital, University of Oxford, Oxford, UK; 7 Present Address: Department of Genetics, Blavatnik Institute, Harvard Medical School, Boston, MA USA

**Keywords:** Pluripotency, Developmental biology, Stem cells, Cellular imaging, Transcriptional regulatory elements

## Abstract

The mouse and human embryo gradually loses totipotency before diversifying into the inner cell mass (ICM, future organism) and trophectoderm (TE, future placenta). The transcription factors TFAP2C and TEAD4 with activated RHOA accelerate embryo polarization. Here we show that these factors also accelerate the loss of totipotency. TFAP2C and TEAD4 paradoxically promote and inhibit Hippo signaling before lineage diversification: they drive expression of multiple Hippo regulators while also promoting apical domain formation, which inactivates Hippo. Each factor activates TE specifiers in bipotent cells, while TFAP2C also activates specifiers of the ICM fate. Asymmetric segregation of the apical domain reconciles the opposing regulation of Hippo signaling into Hippo OFF and the TE fate, or Hippo ON and the ICM fate. We propose that the bistable switch established by TFAP2C and TEAD4 is exploited to trigger robust lineage diversification in the developing embryo.

## Abstract (web summary)

Here the authors identify the transcription factors TFAP2C and TEAD4 as a bistable switch that reconciles into Hippo ON and OFF states, establishing a composite state at the eight-cell stage and critically regulating lineage diversification.

## Main

**Fig. 1:**
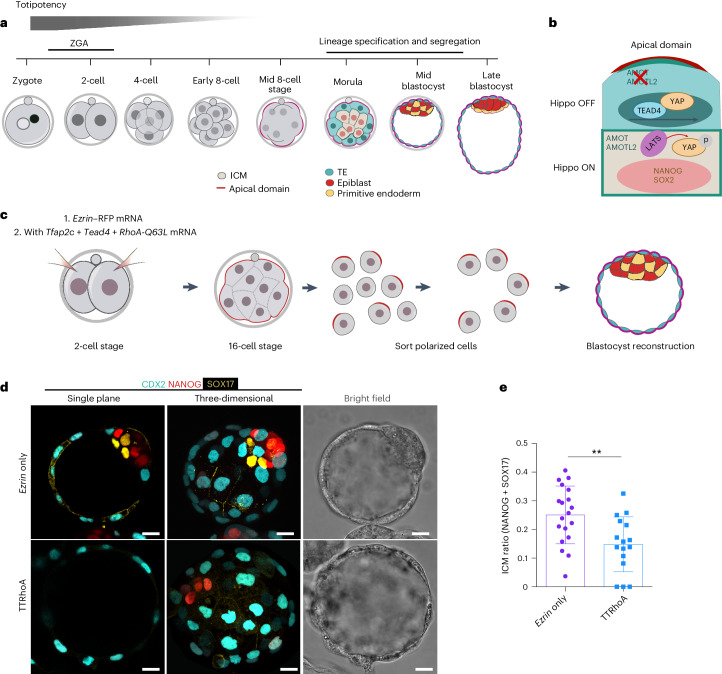
Premature expression of TFAP2C, TEAD4 and activated Rho GTPase are sufficient to advance the first cell fate decision. **a**, A schematic of preimplantation development. ZGA, zygotic genome activation. **b**, A schematic of differential Hippo signalling in TE (top) and ICM (bottom) lineages in the morula stage mouse embryo. **c**, A schematic of blastocyst reconstruction assay. Two-cell stage embryos injected with *Ezrin*–RFP (*Ezrin* only, control) or *Tfap2c* + *Tead4* + *RhoA* mRNA (TTRhoA) were cultured until the early 16-cell stage. Sixteen polarized cells from each genotype were sorted, re-aggregated, cultured until the mid-blastocyst stage and the proportion of ICM examined. **d**, Representative images of the reconstructed blastocysts from *Ezrin*-only or TTRhoA embryos. Embryos were immunostained to reveal CDX2 (TE), NANOG (epiblast) and SOX17 (primitive endoderm). **e**, Quantification of the ratio of ICM from reconstructed blastocysts from *Ezrin*-only or TTRhoA embryos. The ICM ratio is calculated as the number of cells positive for NANOG or SOX17 divided by the total number of cells (positive for CDX2, NANOG or SOX17). Each dot indicates the data point obtained from one embryo. Data shown as mean ± s.e.m. *N* = 19 embryos for EZRIN-only group and *N* = 16 embryos for TTRhoA group. *N* = 2 experiments. **P = 0.0266, two-sided Student’s *t*-test. Scale bars, 15 μm. Source data

In mammals, the highly differentiated sperm and egg fuse to generate a totipotent zygote that gives rise to all the cells in the body and to the extraembryonic tissues. Totipotency gradually decreases during the first few cell divisions (Fig. 1a). At the eight-cell stage, each cell (blastomere) becomes polarized along the outside–inside axis, forming a cap-shape structure on the outside domain called the apical domain^1–5^. Asymmetric segregation of apical domains produces a 16-cell embryo with polar outside cells that will become the trophectoderm (TE, future placenta) and apolar inside cells that will become the inner cell mass (ICM, future epiblast and yolk sac)^6,7^. ICM- and TE-specific transcription factors were found to be co-expressed in blastomeres before lineage diversification^4,8–12^. However, what mechanisms lead to the co-expression of opposite lineage markers and to reconciliation of this bipotency into one of the two fates remain unclear.

Asymmetric segregation of the apical domain results in asymmetric regulation of Hippo signaling (Fig. 1b). In outer cells, the apical domain sequesters and inhibits positive regulators of the Hippo pathway, such as ANGIOMOTIN (AMOT) and ANGIOMOTIN-LIKE 2 (AMOTL2) (ref. ^13^), resulting in a Hippo OFF state and translocation of YAP or TAZ to the nucleus. Nuclear YAP interacts with the transcription factor TEAD4 to induce high expression of TE fate specifiers, including *Cdx2* and *Gata3* and repress pluripotency transcription factors such as *Sox2* (refs. ^8,14–18^). In contrast, the Hippo ON state in ICM cells, which lack an apical domain, leads to YAP phosphorylation, its cytoplasmic retention and subsequent degradation^8,14,16^. High Hippo signaling in the ICM, and the lack of nuclear YAP, TAZ and TEAD4, promote the expression of the pluripotency transcription factors, such as *Sox2* and *Nanog*^17,19^. Polarized outer cells with moderate YAP or TAZ activity that express both the ICM gene *Sox2* and the TE gene *Cdx2* have been observed^17,20^. Hippo signaling in these outer cells disrupts polarization, leading to their repositioning to the ICM^21^. How these ‘conflicted’ outer cells arise and how Hippo signaling itself is established before cell fate specification remain long-standing questions.

*Tead4* and *Tfap2c* are known to be essential for the expression of *Cdx2* after embryo polarization and therefore for formation of the TE lineage at the blastocyst stage^22–25^. *Tead4* knockout (KO) embryos do not form a blastocyst and express the pluripotency factors, OCT4 and NANOG, in all the blastomeres, even the outer ones^24^. Combined depletion of maternal and zygotic TFAP2C also blocks blastocyst formation^22^. Additionally, TFAP2C and the TE specifier GATA3 have been shown to directly couple TE-specific gene induction with suppression of pluripotency^26^. Moreover, embryonic stem cells overexpressing TFAP2C or TEAD4 alone upregulate TE specification genes^14,27^. Thus, TEAD4 and TFAP2C have been recognized as TE fate specifiers in late embryos at the blastocyst stage.

However, TEAD4 and TFAP2C were recently shown to play unique roles also in the early embryo. Their expression is initiated already at the two-cell stage and its gradual increase is required for embryo polarisation at the eight-cell stage^28^. Indeed, we found that co-expressing TFAP2C and TEAD4 with activated RHOA, a GTPase required for apical domain formation^3^, induces precocious embryo polarization at the four-cell stage rather than at the typical eight-cell stage^28^. Moreover, TFAP2C was recently shown to promote the expression of both ICM and TE genes in a bipotency program before lineage diversification^11,12,29^. How the bipotent state is reconciled into two robust lineages, the TE and ICM, remains unclear.

In this Article, we show that TFAP2C and TEAD4 initiate expression of opposite lineage markers to create bipotency and an intermediate level of Hippo signaling in all bipotent blastomeres before cell fate specification. Our results suggest that YAP negative feedback via Hippo signaling^30^ is exploited from the two- to eight-cell stages to diminish totipotency and generate a bistable switch. This switch is ultimately controlled by the presence or absence of the apical domain. Inheritance of the apical domain inhibits intermediate Hippo signaling (Hippo OFF), sustains YAP activity and establishes TE. In contrast, in cells lacking the apical domain, intermediate Hippo signaling becomes Hippo ON, extinguishes YAP activity and establishes ICM. In this way, opposing regulation of Hippo signaling in bipotent blastomeres creates a bistable state that can robustly diversify into the two first lineages.

## Results

### TFAP2C, TEAD4 and RHOA are sufficient to advance cell fate commitment

**Extended Data Fig. 1:**
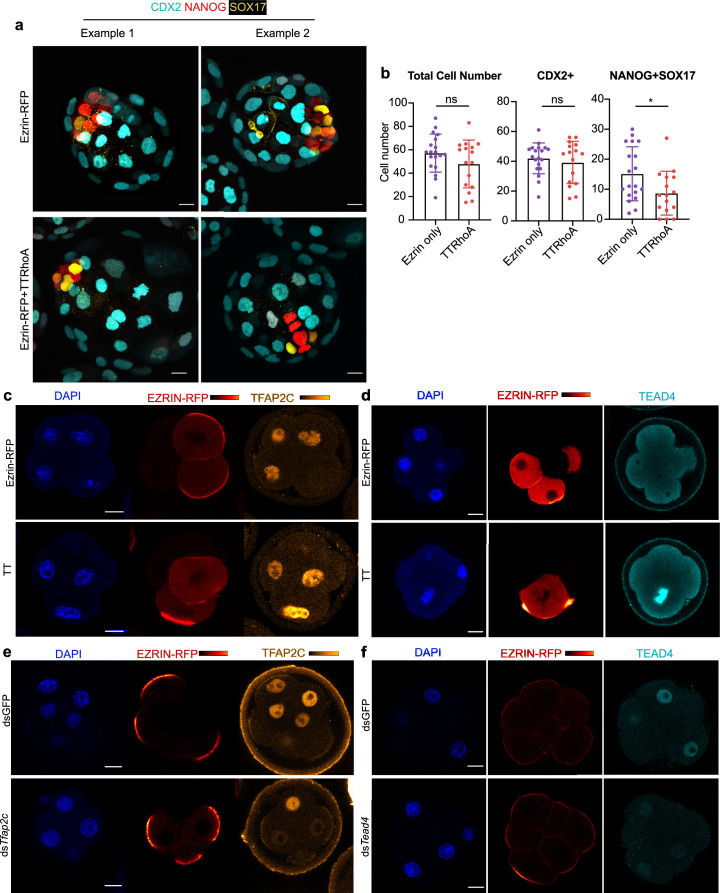
Expression of *Tfap2c*, *Tead4* and activated Rho GTPase are sufficient to advance the first cell fate decision. (**a**) Representative images of the reconstructed blastocysts from Ezrin-only or TTRhoA embryos. Embryos were immunostained to reveal CDX2 (TE), NANOG (epiblast) and SOX17 (primitive endoderm). Experimental procedures were described as in Fig. 1c. (**b**) Quantifications of TE and ICM cell numbers in each reconstructed blastocyst generated from experiment described in Fig. 1c. * p = 0.0276; ns, not significant, Two-sided student’s t test. Each dot indicates the datapoint obtained from one embryo. N = 19 embryos for Ezrin-only group, N = 16 embryos for TTRhoA group. N = 2 experiments. (**c**) Representative images of embryos injected with Ezrin-RFP or with *Tfap2c+Tead4+RhoA* mRNA in one cell of the 2-cell stage, and immunostained with DAPI with TFAP2C at the 4–8 cell stage. (**d**) Representative images of embryos injected with Ezrin-RFP or with *Tfap2c*+*Tead4+RhoA* mRNA at the one cell of the 2-cell stage, and immunostained with DAPI with TEAD4 at the 8-cell stage. (**e**) Representative images of embryos injected with EZRIN-RFP and dsRNA targeting *Tfap2c* at the one cell of the 2-cell stage, and immunostained with DAPI with TFAP2C at the 8- stage. (**f**) Representative images of embryos injected with EZRIN-RFP and dsRNA targeting *Tead4* at both cells of the 2-cell stage, and immunostained with DAPI with TEAD4 at the 8- stage. N = 2 experiments for **c**-**f**. Scale bars, 15 μm. Source data

We previously showed that ectopic expression of TFAP2C, TEAD4 and RHOA (named TTRhoA hereafter) accelerates embryo polarization. Mouse embryos typically polarize at the eight-cell stage; however, embryos expressing TTRhoA polarize already at the four-cell stage^28^. Given that the potential of polarized outer cells to regenerate ICM is progressively lost as blastomeres become committed to the TE fate^20^, we wished to determine whether these three factors are also sufficient to accelerate TE commitment. To test this, we performed a blastocyst reconstruction assay (Fig. 1c). We microinjected both blastomeres of two-cell embryos with EZRIN–red fluorescent protein (RFP) messenger RNA (mRNA) (to mark the apical domain), with or without TTRhoA, using a technique that does not impair development and allows the embryo to develop beyond implantation^31^. Polarized blastomeres were sorted from nonpolarized blastomeres at the mid-16-cell stage, re-aggregated and allowed to develop to the blastocyst stage. We determined the proportion and number of ICM cells relative to total cells in reconstructed blastocysts (Fig. 1c–e and Extended Data Fig. 1a–f). Compared with controls, blastocysts derived from polarized blastomeres expressing TTRhoA had a lower number of ICM cells and therefore a lower ICM ratio (Fig. 1e and Extended Data Fig. 1b). These results indicate that TTRhoA overexpression not only accelerates the timing of embryo polarization^28^ but also accelerates commitment to the TE fate in blastomeres.

### TFAP2C, TEAD4 and RHOA advance apical domain aging and inhibit Hippo signaling

Hippo signaling pathway components at the apical domain in the TE are inactive, leading to nuclear localization of unphosphorylated, active YAP^7,14^. In contrast, active Hippo signaling in the ICM generally triggers YAP phosphorylation (p-YAP), cytoplasmic retention and degradation^32^. Thus, the Hippo pathway negatively regulates YAP activity.

**Fig. 2:**
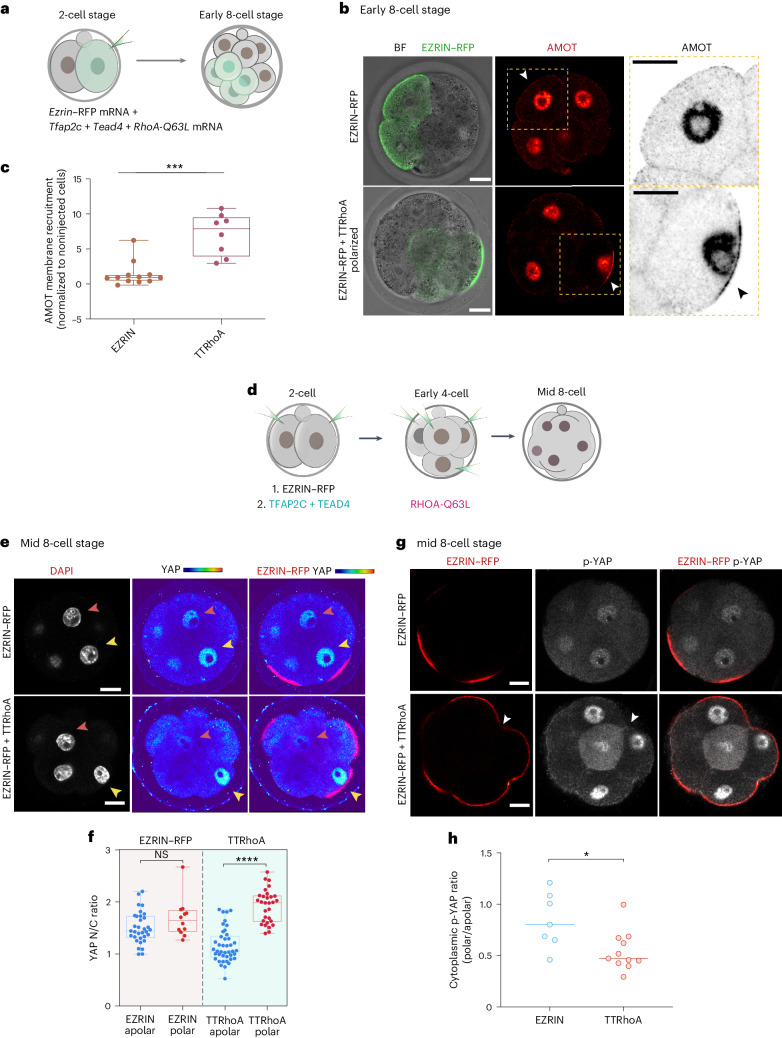
*Tfap2c*, *Tead4* and activated *RhoA* coordinate Hippo inactivation with apical domain formation. **a**, A schematic showing the workflow for experiments in **b** and **c**. **b**, Embryos injected with EZRIN–RFP only (as a control) or TTRhoA mRNAs were analyzed at the early eight-cell stage to reveal EZRIN–RFP and AMOT. The yellow squares indicate the magnified regions. The arrows indicate magnified cells. **c**, Quantifications of apical membrane enrichment of AMOT in cells expressing EZRIN–RFP or with TTRhoA. Data are shown as individual data points with box and whisker plots (lower: 25%; upper: 75%; line: median; and whiskers: min to max). Each dot indicates an analyzed cell. *N* = 12 cells for EZRIN–RFP and *N* = 8 cells for TTRhoA. *N* = 2 experiments. ***P = 0.0002, two-sided Mann–Whitney test. **d**, A schematic of TTRhoA overexpression for experiments shown in **e**–**g**. **e**, Embryos overexpressing EZRIN–RFP only (as a control) or TTRhoA, immunostained at mid eight-cell stage for DNA (DAPI), YAP and EZRIN–RFP. The pink arrows indicate apolar cells and yellow arrows indicate polar cells. Quantifications are shown in **f**. **f**, Quantification of the YAP N/C ratio in the polar or apolar cells of embryos overexpressing EZRIN–RFP only or TTRhoA. Data shown as individual data points with mean, cyan dots indicate polar cells and red dots indicate apolar cells. *N* = 12 embryos for EZRIN–RFP only and *N* = 29 embryos for the TTRhoA group, *N* = 4 experiments, ****P < 0.0001, two-way ANOVA test. YAP N/C ratios between polar and apolar cells are statistically different in the TTRhoA group but not in the EZRIN–RFP only group. **g**, Embryos overexpressing EZRIN–RFP only (as a control) or TTRhoA analyzed at mid eight-cell stage for EZRIN–RFP or p-YAP. The arrows indicate the apolar cells in TTRhoA overexpressing embryos. **h**, Quantification of the cytoplasmic ratio of p-YAP between the polar and apolar cells in embryos overexpressing of EZRIN–RFP only or TTRhoA. Data shown as individual data points with mean indicated by the line. N = 7 embryos for EZRIN–RFP only and *N* = 11 embryos for the TTRhoA group, *N* = 4 experiments and *P < 0.05, Mann–Whitney test. The lower cytoplasmic level of p-YAP in polar versus apolar cells in TTRhoA embryos versus controls. For all quantifications, data are shown as individual data points with mean. Scale bars, 15 μm. Source data

**Extended Data Fig. 2:**
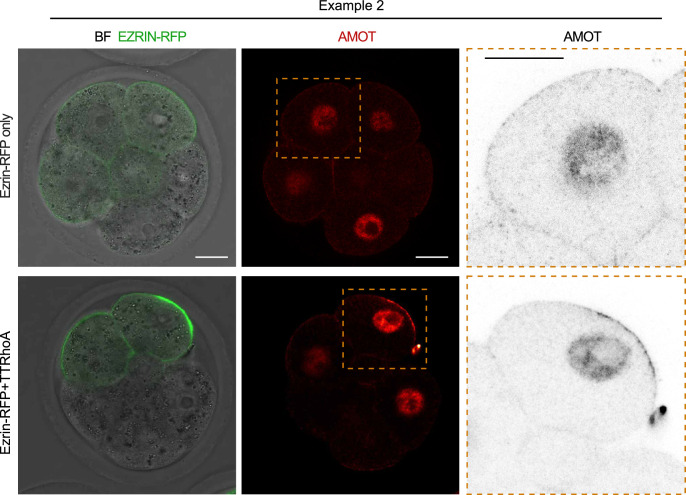
AMOT protein is tethered to the prematurely formed apical domain by the expression of *Tfap2c*, *Tead4*, and activated Rho GTPase. Embryos injected with Ezrin-RFP only (as a control) or TTRhoA mRNAs at one cell of the 2-cell stage and were analysed at the early 8-cell stage to reveal EZRIN-RFP and AMOT. Yellow squares indicate the magnified regions. Arrows indicate magnified cells. Experimental procedures were described as in Fig. 2a. N = 2 experiments. Scale bars, 15 μm.

To determine how the acceleration of polarization and TE commitment in TTRhoA blastomeres affects Hippo signaling, we expressed the apical marker, EZRIN–RFP, with or without TTRhoA in one blastomere at the late two-cell stage. The uninjected blastomere served as a noninjection control (Fig. 2a). We found that none of the EZRIN–RFP blastomeres in control early eight-cell embryos displayed an apical domain^3,5^, whereas about 30% of the blastomeres in EZRIN–RFP + TTRhoA had an apical domain already at the early eight-cell stage^28^ (Fig. 2b,c and Extended Data Fig. 2).

**Extended Data Fig. 3:**
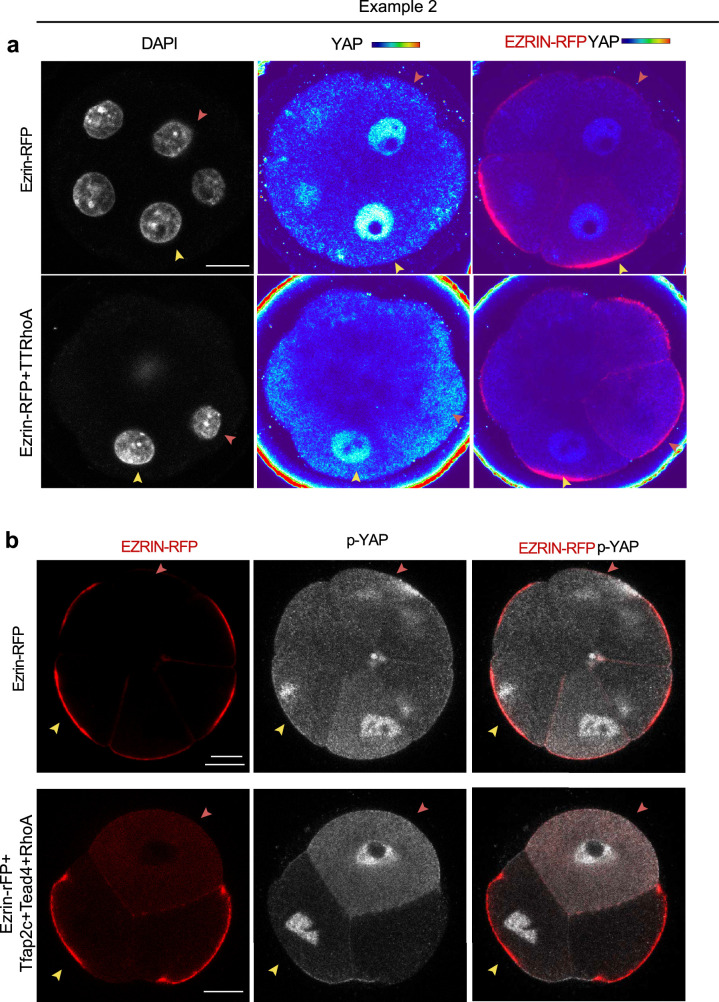
Expression of *Tfap2c*, *Tead4* and activated Rho GTPase promotes nuclear localisation of YAP in polar cells. (**a**) Representative images of embryos injected with mRNA encoding EZRIN-RFP alone (control) or with TTRhoA mRNA as described in Fig. 2a and immunostained at the mid 8-cell stage to reveal the localisation of EZRIN-RFP and YAP. (**b**) Representative images of embryos injected with mRNA encoding EZRIN-RFP alone (control) or with TTRhoA mRNA as described in Fig. 2d, and immunostained at the mid 8-cell stage to reveal the localisation of EZRIN-RFP and p-YAP. Yellow arrows indicate polarised cells, red arrows indicate unpolarised cells. N = 4 experiments. Scale bars, 15 μm.

To investigate the consequences of precocious apical domain formation on Hippo signaling, we wished to determine YAP localization in TTRhoA embryos versus controls. To this end, we upregulated expression of TT at the two-cell stage, introduced activated RHOA at the four-cell stage and examined embryos at the mid eight-cell stage (Fig. 2d). Both EZRIN–RFP-only and TTRhoA mid eight-cell stage embryos had a mixture of polarized and unpolarized cells, with more polarized cells in TTRhoA embryos than in controls (Fig. 2e–h). In control embryos, the polarized and unpolarized blastomeres displayed similar nuclear-to-cytoplasmic (N/C) ratios of YAP (Fig. 2e,f and Extended Data Fig. 3a). In contrast, in TTRhoA mid eight-cell embryos, the N/C ratios of YAP were higher in polar versus apolar blastomeres (Fig. 2g,h and Extended Data Fig. 3b). Moreover, the levels of cytoplasmic p-YAP were lower in polar versus apolar blastomeres of TTRhoA versus control embryos (Fig. 2h). We often detected nuclear p-YAP in blastomeres, which has been observed in other contexts, particularly in sparsely plated cells^33,34^ (Discussion). Overall, these data suggest that Hippo signaling diminishes after polarization of TTRhoA embryos, which indicates that TTRhoA not only advance polarization timing but also enhance the function of the apical domain, resulting in reduced Hippo signaling in blastomeres.

### TFAP2c and TEAD4 activate TE and ICM genes to create a bipotent state

**Extended Data Fig. 4:**
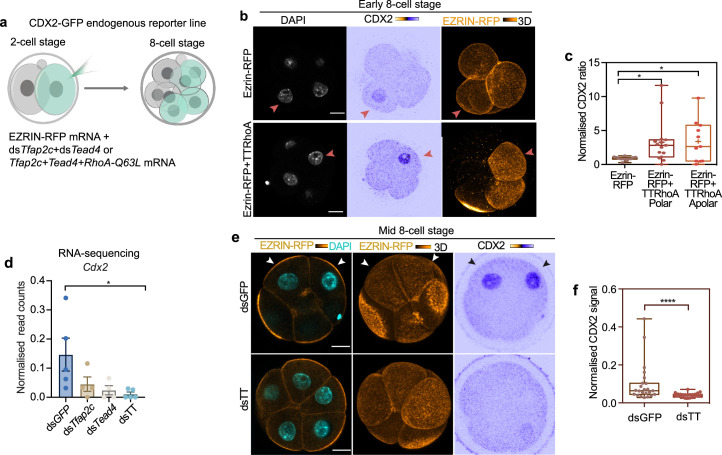
*Tfap2c* and *Tead4* regulates the expression of *Cdx2* before polarisation. (**a**) Schematic of workflow for experiments in (b-c, e-f). (**b**) Embryos injected with Ezrin-only or also with TTRhoA and analysed at early 8-cell stage for DAPI, CDX2 and EZRIN-RFP. Arrows indicate an injected cell. Quantification shown in c. (**c**) Quantification of normalised CDX2 nuclear signal intensity in Ezrin-only (polar and apolar) cells, TTRhoA polar cells and TTRhoA apolar cells. (Data shown as individual data points with Box and Whisker plots (lower: 25%; upper: 75%; line: median; whiskers: min to max). Each dot indicates an analysed cell. N = 13 embryos for EZRIN-RFP only; n = 15 embryos for TTRhoA group. *p < 0.05, one-way ANOVA test. (**d**) RNA-sequencing analysis of *Cdx2* expression level at the 8-cell stage in embryos injected with dsRNA targeting GFP, *Tfap2c*, *Tead4* or *Tfap2c* and *Tead4* (dsTT) at the zygote stage. N = 5 samples for dsGFP and dsTT; n = 4 samples for ds*Tfap2c* and ds*Tead4*. Data is shown as mean ± S.E.M. *p = 0.0159, Kruskal-Wallis test. (**e**) Representative images of CDX2 expression in cells injected with Ezrin-only or also with dsTT at the mid 8-cell stage. Quantification is shown in panel f. (**f**) Quantification of normalised CDX2 nuclear signal intensity. For the normalisation, the nuclear signal of CDX2 was normalised against DAPI, and then the CDX2/DAPI ratio of injected cells were normalised against the value of non-injected cells. Data shown as individual data points with Box and Whisker plots (lower: 25%; upper: 75%; line: median; whiskers: min to max). N = 13 embryos for EZRIN-RFP only; n = 15 embryos for dsTT group. ****p < 0.0001, Mann-Whitney test. Normalisation is as in d. Scale bars, 15 μm. Source data

We previously found that TTRhoA embryos had precocious expression of the TE specifier *Cdx2* (ref. ^28^). To determine whether this is related to advanced polarization, we microinjected EZRIN–RFP with or without TTRhoA in one blastomere of two-cell stage embryos (Extended Data Fig. 4a). These embryos express an endogenous CDX2–green fluorescent protein (GFP) reporter^35^, which showed low CDX2–GFP expression in blastomeres of control eight-cell embryos (Extended Data Fig. 4b) as expected^36,37^. TTRhoA overexpression led to an upregulation of CDX2–GFP not only in polarized cells, but also in apolar cells (Extended Data Fig. 4b,c). Consistent with a previous study^22^, *Tfap2c* depletion led to reduced *Cdx2* levels in mid eight-cell stage embryos by RNA sequencing (Extended Data Fig. 4d). Moreover, depletion of both *Tead4* and *Tfap2c* exacerbated the decrease in *Cdx2* mRNA levels (Extended Data Fig. 4d). Analysis of CDX2–GFP reporter embryos revealed that depletion of *Tfap2c* and/or *Tead4* in two-cell embryos significantly reduced the GFP levels in apolar blastomeres of eight-cell embryos, consistent with the RNA-sequencing data (Extended Data Fig. 4d–f). Thus, *Tfap2c* and *Tead4* each promote expression of the TE marker *Cdx2* even before lineage specification, in both polar and apolar cells.

**Fig. 3:**
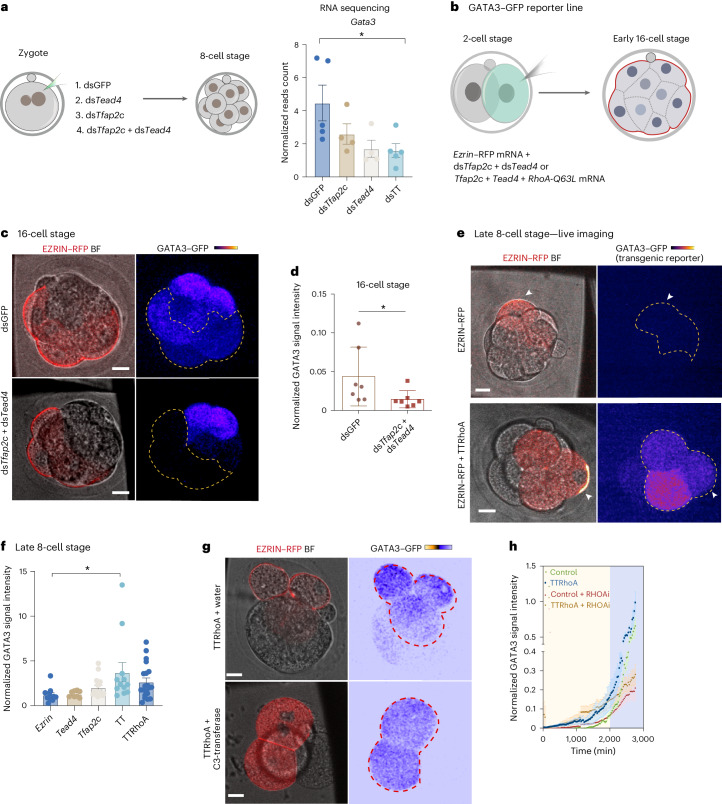
*Tfap2c* and *Tead4* regulate the expression of *Gata3* before polarization. **a**, RNA-sequencing analysis of *Gata3* expression level at the eight-cell stage in embryos injected with dsGFP, ds*Tfap2c*, ds*Tead4* or ds*Tfap2c* + ds*Tead4*. *N* = 5 samples for ds*GFP* and ds*Tfap2c* + ds*Tead4* and *N* = 4 samples for ds*Tfap2c* and ds*Tead4*. Data are shown as mean ± s.e.m. *P < 0.05, Kruskal–Wallis test. *N* = 2 collections. **b**, A schematic of workflow for experiments in **c**–**h**. One blastomere of the two-cell stage embryo was injected with mRNA encoding *Ezrin* only (as a control), or also with dsRNA targeting *Tfap2c* and *Tead4*, or also with *TTRhoA* mRNA. **c**, Representative images of GATA3–GFP expression level in 8–16-cell stage embryos injected with the indicated dsRNA as described in **b**. Quantifications are shown in **d**. *P < 0.05, Mann–Whitney test. **d**, Quantification of the level of GFP in control and embryos injected with dsRNA targeting *Tfap2c* and *Tead4*. *P = 0.0262, Mann–Whitney test. Data are shown as mean ± s.e.m. *N* = 7 embryos for the *Ezrin*-only group and *N* = 7 embryos for the ds*Tfap2c* + ds*Tead4* group. *N* = 2 experiments. **e**, Representative images of GATA3–GFP transgenic late eight-cell embryos, after injection with EZRIN only or TTRhoA, as described in **b**. The arrows indicate an injected cell. The number of embryos and quantifications shown in **f**. BF, bright field. **f**, Quantifications of normalized GATA3–GFP signal intensity in the indicated overexpression conditions. For normalization, GFP signal in injected cells were normalized against the noninjected cells. Data are shown as mean ± s.e.m. The numbers indicate the number of embryos analyzed. *P = 0.0218, one-way ANOVA test. **g**, Representative images of GATA3–GFP expression in embryos injected with EZRIN–RFP mRNA and TTRhoA, as indicated in **b**, and treated with water (control) or C3-transferase (RhoA inhibitor) at the late eight-cell stage. Quantifications are shown in **h**. **h**, A time course of t**h**e normalized GATA3–GFP signal intensity in cells overexpressing EZRIN–RFP only (control), or also exposed to TTRhoA, RhoA inhibitor or TTRhoA + RhoA inhibitor. Data are shown as mean ± s.e.m. *n* = 7 embryos for each group. The yellow region indicates the early stages of developmental when Gata3 expression is insensitive to RhoA activity (before the 16-cell stage) and the purple region indicates RhoA-sensitive stages (after the 16-cell stage). Scale bars, 15 μm. Source data

**Extended Data Fig. 5:**
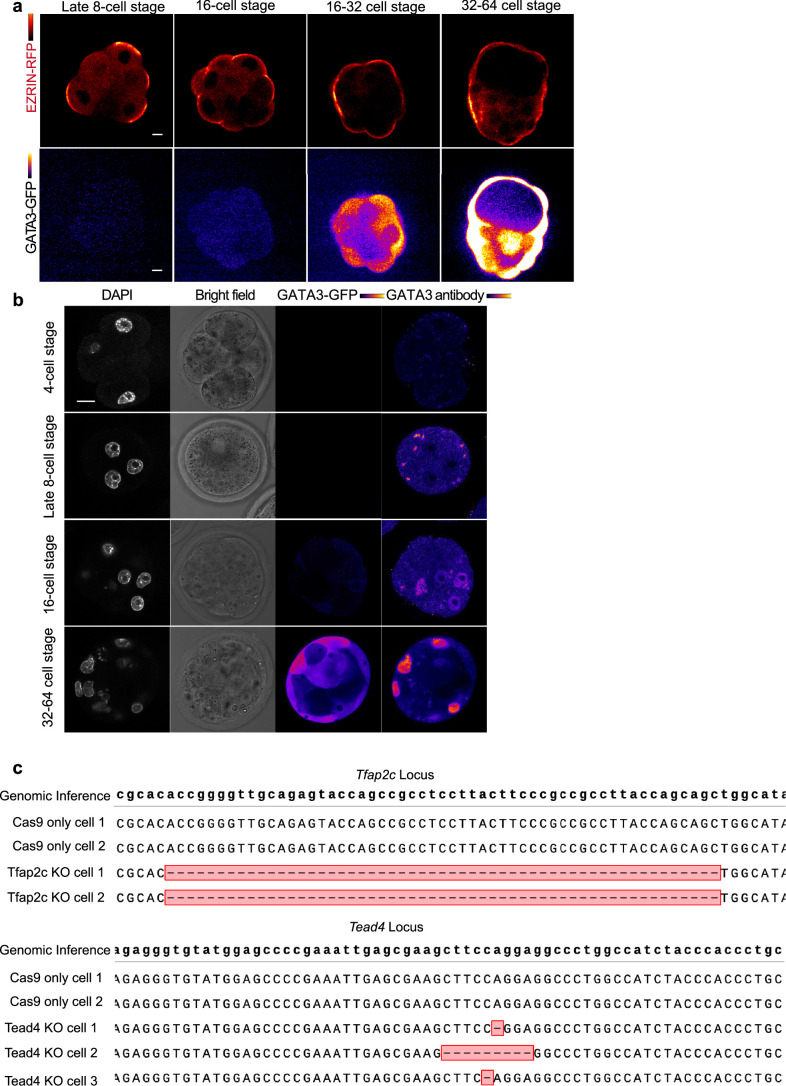
Expression profile of the GFP signal in GATA3-GFP transgenic mouse line. (**a**) Snapshots of GATA3-GFP transgenic embryos injected with EZRIN-RFP at different developmental stages. GFP signal was undetectable until 16- cell stage, when it was expressed in all cells. Segregation of GFP to the outer polarised cells is observed from 16–32 cell stage. n = 35 embryos examined. Scale bars, 15 μm. (**b**) Representative images of 4-, late 8-, 16- and 32–64 cell stage embryos carrying GATA3-GFP transgene to immunostain with DAPI and GATA3. A similar expression profile in time and space is detected between the signals yielded from GATA3-GFP transgene reporter and GATA3 antibody. N = 2 embryos for each stage. Scale bars, 15 μm. (**c**) Single cell genotyping of cells that show high (Cas9 only group) and low (Cas9+sgRNAs) levels of TFAP2C and TEAD4 protein. Criteria for cell grouping is as previously described.

The TE marker *Gata3* is expressed independently of *Cdx2* (ref. ^15^) and our RNA-sequencing data revealed that depletion of *Tfap2c* and *Tead4* also reduced the expression of *Gata3* in eight-cell embryos (Fig. 3a). Embryos expressing a GFP reporter driven by the *Gata3* promoter^38^ displayed GFP expression in all cells at the early 16–32 cell stage, followed by preferential expression in outer polar cells at the 32–64 cell stage (Extended Data Fig. 5a,b) as expected^15^. Notably, depleting *Tfap2c* and *Tead4* at the two-cell stage strongly abrogated *Gata3* expression in both polar and apolar blastomeres at the early 16-cell stage (Fig. 3b–d). Moreover, GATA3 was prematurely expressed in TTRhoA embryos (2.7-fold upregulation at the late eight-cell stage; Fig. 3e,f). Notably, embryos expressing *Tfap2c* and *Tead4* alone, as well as TTRhoA embryos treated with the Rho inhibitor C3-transferase^39^, showed polarity-independent induction of GATA3 at the eight-cell stage but not after (Fig. 3g,h). Thus, *Tfap2c* and *Tead4* also each promote expression of the TE marker *Gata3* even before lineage specification.

**Fig. 4:**
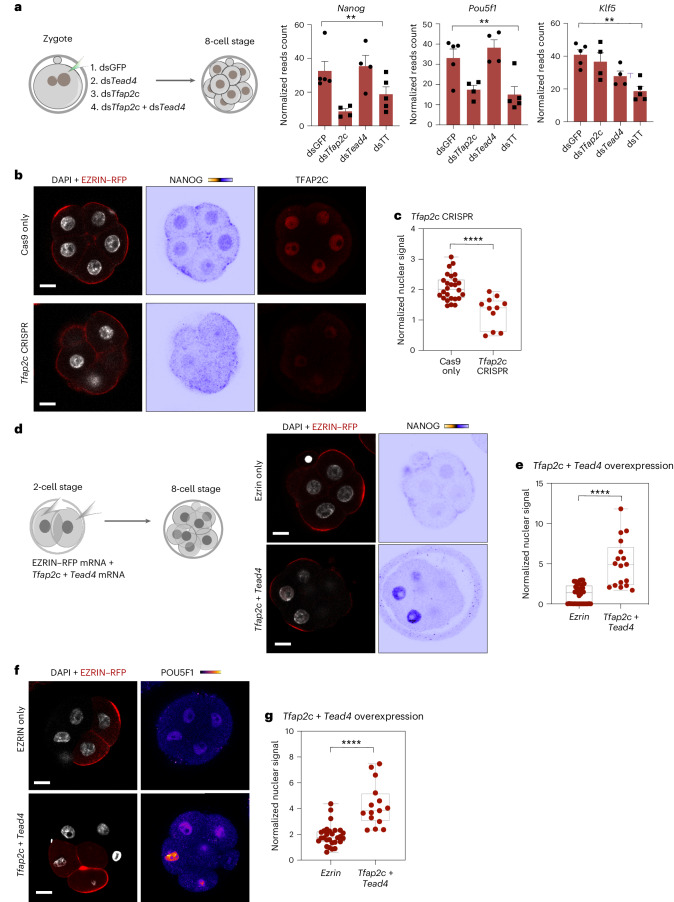
*Tfap2c* and *Tead4* regulate the expression of ICM specifiers before polarization. **a**, The expression of *Nanog*, *Pou5f1* (*Oct4*) and *Klf5* by bulk RNA sequencing in the indicated conditions. **P = 0.0042 for *Nanog*, *P* = 0.0021 for *Pou5f1* and *P* = 0.0019 for *Klf5*, Kruskal–Wallis test. *N* = 5 samples for ds*GFP* and ds*Tfap2c* + ds*Tead4* and *N* = 4 samples for ds*Tfap2c* and ds*Tead4*. *N* = 2 collections. Data are shown as mean ± s.e.m. **b**, Representative images of embryos injected with Cas9 mRNA or with gRNAs targeting *Tfap2c* gene locus, to fix at the mid eight-cell stage and stain for NANOG and TFAP2C. The quantification is shown in **c**. **c**, Quantification of NANOG expression in Cas9-only (control) or *Tfap2c*-depleted cells by CRISPR–Cas9 shown in **b**. ****P < 0.0001, two-sided Student’s *t*-test. *N* = 27 embryos for the Cas9-only group and *N* = 10 embryos for *Tfap2c* KO embryos. *N* = 2 experiments. Data are shown as individual data points with box and whisker plots (bottom: 25%; upper: 75%; line: median; whiskers: min to max). **d**, Representative images of embryos injected with EZRIN–RFP mRNA alone or with *Tfap2c* and *Tead4* mRNA, and visualized NANOG expression at the mid eight-cell stage. The embryos were injected at the two-cell stage and fixed at the mid eight-cell stage. **e**, Quantification of NANOG protein levels in conditions showing in **d**. *N* = 39 cells for the EZRIN–RFP group and *N* = 17 cells for the *Tfap2c* + *Tead4* group. *N* = 2 experiments. ****P < 0.0001, Mann–Whitney test. Data are shown as individual data points with box and whisker plot (bottom: 25%; upper: 75%; line: median; whiskers: min to max). **f**, Representative images of embryos injected with EZRIN–RFP mRNA alone or with *Tfap2c* and *Tead4* mRNA at one cell of the two-cell stage and fixed at the early to mid eight-cell stage, and visualized POU5F1 (OCT4) expression at the mid eight-cell stage. **g**, Quantification of OCT4 protein levels in cells from conditions shown in **f**. *N* = 28 cells for the *Ezrin*–RFP group and *N* = 15 cells for the *Tfap2c* + *Tead4* group. *N* = 2 experiments and ****P < 0.0001, Mann–Whitney test. Data are shown as individual data points with box and whisker plots (bottom: 25%; upper: 75%; line: median; whiskers: min to max). Scale bars, 15 μm. Source data

We also found that *Tfap2c* leads to the upregulation of the ICM specifiers, *Nanog* and *Oct4*, which are expressed in all blastomeres at the eight-cell stage and become restricted to the ICM at the mid-blastocyst stage^40–44^. We found that the depletion of TFAP2C, but not TEAD4, led to downregulation of *Oct4* (Fig. 4a–c), consistent with previous indications^29,45,46^. Moreover, we found that *Nanog* expression was downregulated upon the depletion of TFAP2C but not TEAD4 (Fig. 4a–c). In addition, co-expression of TFAP2C- and TEAD4-induced upregulation of *Nanog* and *Oct4* at the eight-cell stage (Fig. 4d–g). These data further suggest that TFAP2C activates ICM genes in bipotent cells, in agreement with a recent study showing that TFAP2C binds and activates not only TE genes but also some early ICM genes^12^.

These data suggest temporally distinct functions for *Tead4* and *Tfap2c* during development. Before lineage specification, *Tfap2c* and *Tead4* promote biopotency through inducing the expression of ICM and TE markers and prime both fates. After specification, *Tead4* and *Tfap2c* promote the TE fate^20^.

### TFAP2c and TEAD4 promote the expression of Hippo regulators and Hippo signaling

**Fig. 5:**
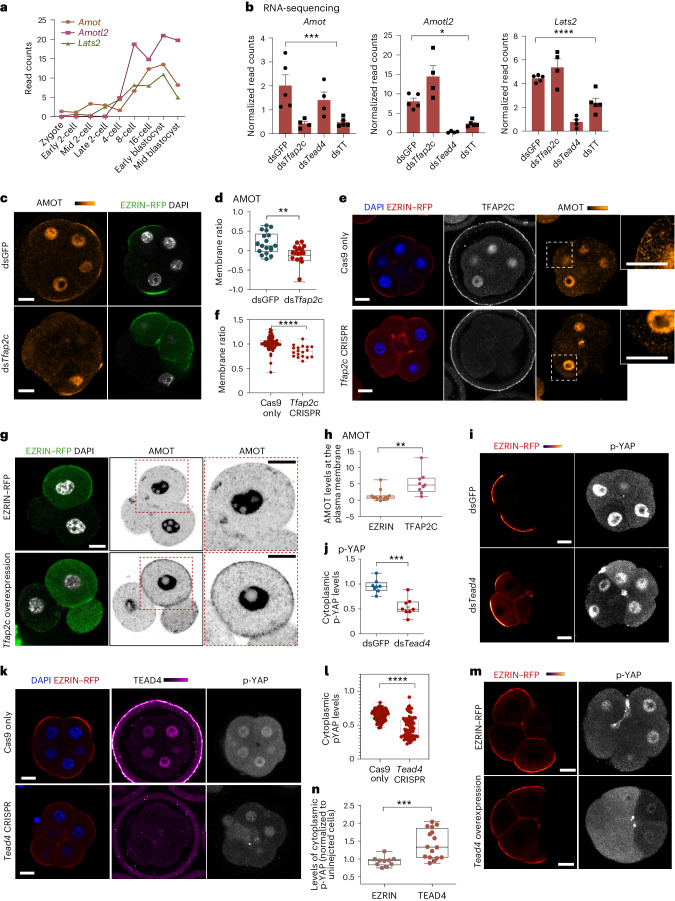
*Tfap2c* and *Tead4* regulate *Amot*, *Amotl2* and *Lats2* and activate Hippo signaling. **a**, The expression profile of *Amot*, *Amotl2* and *Lats2*, data obtained from Deng et al.^47^ **b**, The expression of *Amot* (***P = 0.0052, Kruskal–Wallis test), *Amotl2* (*P = 0.0115, one-way ANOVA) and *Lats2* (****P < 0.0001, Kruskal–Wallis test) by bulk RNA sequencing in the indicated conditions. The expression level is shown as mean ± s.e.m. *N* = 5 samples for ds*GFP* and ds*Tfap2c* + ds*Tead4* and *N* = 4 samples for ds*Tfap2c* and ds*Tead4*. *N* = 2 collections. **c**, Late eight-cell embryos injected with EZRIN–RFP + dsGFP (control) or EZRIN–RFP + ds*Tfap2c* in half embryo and immunostained AMOT, EZRIN–RFP and DNA (DAPI). BF, bright field. **d**, Quantification of plasma membrane-localized AMOT as in **c**. *N* = 17 cells for EZRIN–RFP and *N* = 14 cells for ds*Tfap2c*. *N* = 2 experiments. **P = 0.0087, two-sided Mann–Whitney test. **e**, Mid-eight-cell embryos injected with Cas9 mRNA or with *Tfap2c* sgRNAs stained with TFAP2C and AMOT. **f**, Quantification of membrane AMOT as in **e**. *N* = 62 cells for Cas9 only and *N* = 18 cells for *Tfpa2c* CRISPR. *N* = 2 experiments. ****P < 0.0001, two-sided Mann–Whitney test. **g**, Late four-cell embryos overexpressing EZRIN–RFP or with *Tfap2c* in half embryo immunostained with AMOT, EZRIN–RFP and DNA (DAPI). **h**, Quantification of membrane AMOT as in **k**. *N* = 11 embryos for EZRIN and *N* = 9 embryos for the *Tfap2c* group. *N* = 2 experiments. **P = 0.0013, two-sided Mann–Whitney test. **i,** Late eight-cell embryos **i**njected with dsRNA targeting GFP (control) or *Tead4* dsRNA in half embryo and immunostained EZRIN–RFP and p-YAP. **j**, Quantification of cytoplasmic p-YAP levels as in **g**. ***P < 0.001, Mann–Whitney test. *N* = 8 embryos for dsGFP and *N* = 8 embryos for ds*Tead4*. *N* = 2 experiments. **k**, Mid eight-cell embryos injected with Cas9 mRNA or with *Tead4* sgRNAs stained with TEAD4 and p-YAP. **l**, Quantification of cytoplasmic p-YAP levels as in **i**. *N* = 87 cells for Cas9 and *N* = 68 cells for *Tead4* CRISPR. *N* = 2 experiments. ****P < 0.0001, two-sided Mann–Whitney test. **m**, Mid eight-cell embryos overexpressing EZRIN–RFP or with *Tead4* in half embryo immunostained EZRIN–RFP and p-YAP. **n**, Quantification of cytoplasmic p-YAP as in **m**. *N* = 10 embryos for EZRIN and N = 16 embryos for the *Tead4* group. *N* = 2 experiments. ***P = 0.0002, two-sided Mann–Whitney *t*-test. For **d**, **f**, **h**, **j**, **l** and **n**, data are shown as individual data points with box and whisker plots (bottom: 25%; upper: 75%; line: median; whiskers: min to max). Scale bars, 15 μm. Source data

**Extended Data Fig. 6:**
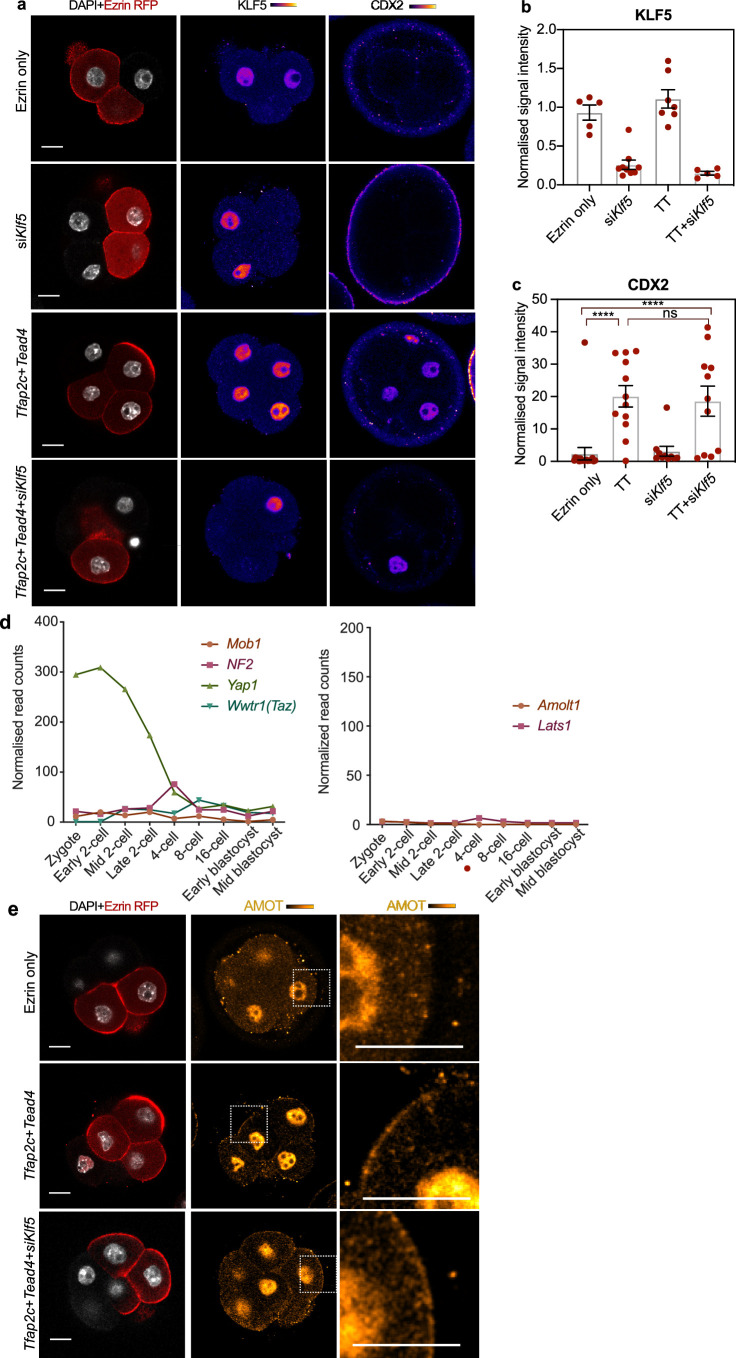
*Tfap2c* and *Tead4* activate the expression of TE and ICM cell fate regulators and Hippo components independent of *Klf5.* (**a**) Representative images of embryos microinjected with EZRIN-RFP mRNA alone, or with *Tfap2c*+*Tead4* mRNA, or *Klf5* siRNA, or *Tfa2pc*+*Tead4* mRNA+*Klf5* siRNA in one cell of the 2-cell stage, and to immunostain for KLF5, CDX2 and DAPI. (**b**) Quantification of KLF5 protein levels in conditions showing in a. KLF5 siRNA effectively eliminated KLF5 expression at the protein level, Tfap2c and Tead4 overexpression had no effect on KLF5’s expression. (**c**) Quantification of CDX2 protein levels in conditions shown in a. Tfap2c and Tead4 expression induced significant upregulation of CDX2 expression at the protein level regardless of the depletion of KLF5. ****p < 0.0001, ns, not significant, Kruskal-Wallis’ test. For b and c, data are shown as bar chart with mean ± SEM and individual data points. N = 19 embryos from Ezrin only group; N = 12 embryos from Tfap2c+Tead4 group; =10 embryos from siKlf5 group; N = 11 embryos from Tfap2c+Tead4+siKlf5 group. N = 2 experiments. (**d**) The expression profile of *Nf2*, *Mob1*, *Yap1*, *Wwtr1*, *Amotl1* and *Lats1* throughout preimplantation development. Data obtained from Deng et al.^47^. (**e**) Representative images of embryos microinjected with EZRIN-RFP mRNA alone, or with *Tfap2c*+*Tead4* mRNA, or *Tfa2pc*+*Tead4* mRNA+*Klf5* siRNA in one cell of the two-cell stage, and immunostained for AMOT and DAPI. Scale bars, 15 μm. Source data

We next wished to determine when Hippo signaling is initiated during development. To this end, we examined a previously published RNA-sequencing dataset^47^ to analyze the expression of Hippo signaling components. We found that *Amot*, *Amotl2* and *Lats2* were not expressed in the zygote but increased at least tenfold from the 2–4-cell stage to the blastocyst stage (Fig. 5a and Extended Data Fig. 6d), concomitant with increased expression of *Tfap2c* and *Tead4* (ref. ^28^). To determine whether *Tfap2c* and *Tead4* control the induction of these Hippo pathway components, we simultaneously depleted TFAP2C and TEAD4 by RNA interference (RNAi) and clustered regularly interspaced short palindromic repeats (CRISPR), which reduced the levels of all three transcripts at the eight-cell stage^28^ (Fig. 5b and Extended Data Fig. 5c).

We found that RNAi-mediated depletion of TFAP2C in zygotes reduced *Amot* expression by 79% in eight-cell embryos. To confirm this result, we also carried out CRISPR-mediated depletion of TFAP2C, which we previously showed led to efficient gene editing of the *Tfap2c* locus^28^. We found that both RNAi- and CRISPR-mediated depletion of TFAP2C reduced the levels of AMOT protein in late eight-cell embryos (Fig. 5c–f). In particular, the relative levels of active AMOT at the plasma membrane versus cytoplasm were reduced by 21.4% and 71% upon depletion of TFAP2C by RNAi and CRISPR^8,16,28^ (Fig. 5c–f). In contrast, overexpression of TFAP2C alone in two-cell embryos led to increased levels of active AMOT at the plasma membrane (Fig. 5g,h).

In addition, RNAi-mediated depletion of *Tead4* in zygotes reduced the expression of *Amotl2* and *Lats2* by 97% and 83%, respectively (Fig. 5b). Consistent with reduced LATS2 activity and diminished Hippo signaling^48^, TEAD4 depletion by RNAi or CRISPR decreased the levels of cytoplasmic p-YAP by 46% and 52.8% in eight-cell embryos, respectively (Fig. 5i–l). Moreover, overexpression of *Tead4* led to increased levels of p-YAP in the cytoplasm (Fig. 5m,n), consistent with elevated Hippo signaling. Yet we previously found that TEAD4 overexpression increases the N/C ratio of YAP before blastomere polarization^28^. In agreement with this, nuclear localization of active YAP has been shown in other contexts to initiate a negative feedback loop via Hippo-mediated phosphorylation of cytoplasmic YAP^30^. These results indicate that *Tfap2c* and *Tead4* each positively regulate Hippo signaling by promoting the expression of Hippo pathway components before polarization, but also negatively regulate Hippo signaling by promoting apical domain formation and aging (Fig. 2). The overall result is intermediate Hippo signaling and the bipotent TE/ICM fate.

Before lineage specification, blastomeres express the transcription factor Kruppel-like factor 5 (KLF5), which directly induces both ICM and TE specification genes^49^. Although *Klf5* was downregulated in blastomeres depleted for *Tfap2c* and *Tead4* (Fig. 4a), it was not elevated along with the ICM and TE lineage specifiers in embryos with ectopic expression of TFAP2C and TEAD4 (Extended Data Fig. 6a–c). To determine whether *Klf5* is required for the *Tfap2c-* and *Tead4-*dependent regulation of lineage markers, we depleted KLF5 in embryos overexpressing TFAP2C and TEAD4. RNAi-mediated depletion of KLF5 effectively eliminated KLF5 protein, but did not affect upregulation the TE marker CDX2 nor did it compromise the ICM feature of increased levels of active AMOT at the plasma membrane (Extended Data Fig. 6). These data suggest that *Tfap2c* and *Tead4* regulate *Klf5* and bipotency independently.

Our results overall show that *Tfap2c* promotes the expression of the TE regulators *Cdx2* and *Gata3*, the ICM regulators *Nanog* and *Oct4*, and the Hippo regulator *Amot* in bipotent cells*; Tead4* similarly promotes the expression of *Cdx2* and *Gata3* and the Hippo regulators *Amotl2* and *Lats2* in bipotent cells. As a consequence, bipotent blastomeres have a sum of intermediate Hippo signaling and display both active nuclear YAP and inactive cytoplasmic p-YAP.

### Potential role of TFAP2C in the first cell fate decision in human embryos

**Fig. 6:**
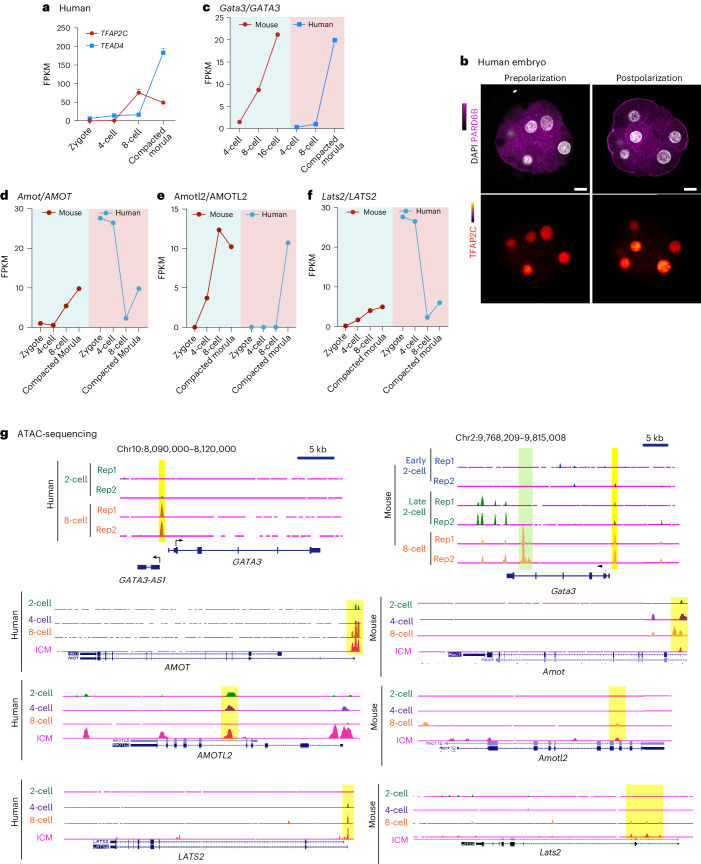
*TFAP2C* regulates gene expression prior to cell compaction in the early human embryo and the model. **a**, The mRNA expression profiles of *TFAP2C* and *TEAD4* in preimplantation human embryos. Data retrieved from Stirparo et al., 2018 (ref. ^50^). Data are shown as mean ± s.e.m. **b**, Human embryos before and after polarization were fixed and stained for PARD6 and TFAP2C. *N* = 4 embryos were examined. **c**–**f**, mRNA expression profile of *Gata3/GATA3* (**c**), *Amot/AMOT* (**d**), *Amotl2/AMOTL2* (**e**) and *Lats2/LATS2* (**f**) in preimplantation mouse and human embryos. Data retrieved from Stirparo et al., 2018 (ref. ^50^). **g**, The University of California, Santa Cruz browser view showing accessible chromatin regions in *Gata3/GATA3* and *Amot/AMOT*, *Amot*l2*/AMOTL2* and *Lats2/LATS2* loci in the mouse and human embryos at different stages, determined from ATAC-sequencing data. Mouse data retrieved from Wu et al.^61^. Human data retrieved from Wu et al.^55^. Scale bars, 15 μm. Source data

Mammalian development at the preimplantation stage is evolutionarily conserved in both gross morphology and expression of the representative lineage markers^50–54^. To determine whether the gene expression network that we identified here in the mouse embryo is conserved in humans, we first examined published RNA-sequencing datasets. We found that the expression of *TFAP2C* increases from the four-cell stage whereas the expression of *TEAD4* increases from the eight-cell stage in the human embryo^55^ (Fig. 6a). We confirmed this early presence of TFAP2C before cell polarization in human embryos by immunostaining (Fig. 6b). ATAC-sequencing data also suggest that genomic binding sites for *TFAP2C*, but not *TEAD4*, are highly accessible at the precompaction stage of the human embryo^55^.

**Extended Data Fig. 7:**
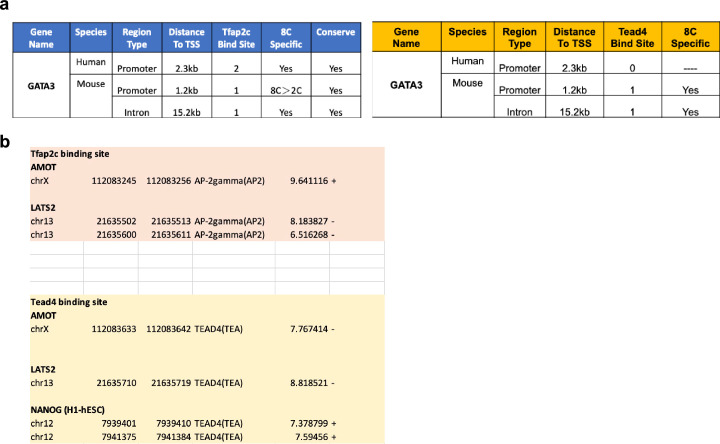
Accessible TFAP2C binding sites are conserved between mouse and human genomic locus. (**a**) DNA sequence of the enhancer, promoter and the intron regions of mouse and human *Gata3/GATA3* locus was analysed and accessible TFAP2C/TEAD4 binding sites were predicted. Sites with DNA sequence homology above 50% were considered as conserved. (**b**) DNA sequence of the enhancer, promoter and the intron regions of mouse and human and *Amot/AMOT*, *Amotl2/AMOTL2*, *Lats2/LAST2 were* analysed and accessible TFAP2C/TEAD4 binding sites were predicted. DNA sequence homology above 50% were considered as conserved.

When we compared the RNA-sequencing and ATAC-sequencing datasets between mouse and human embryos, we found that the TE transcription factor *Gata3/GATA3* and Hippo components *Amot/AMOT*, *Amotl2/AMOTL2* and *Lats2/LATS2*, which are regulated by *Tead4* and *Tfap2c* in mouse embryos^28^ shared similar zygotic patterns of expression to that of the human embryo (Fig. 6c–g). We identified specific *cis*-regions near these genes that become accessible only around the four- or eight-cell stage in the human embryo. These *cis*-regions contained both TFAP2C and TEAD4 consensus sequences in the mouse genome, but only TFAP2C consensus sequences were detected in the human genome (Extended Data Fig. 7). It will be of interest to determine whether TFAP2C regulates bipotency and a bistable switch in blastomeres of the human embryo, in addition to its known roles in polarization and lineage segregation.

## Discussion

**Fig. 7:**
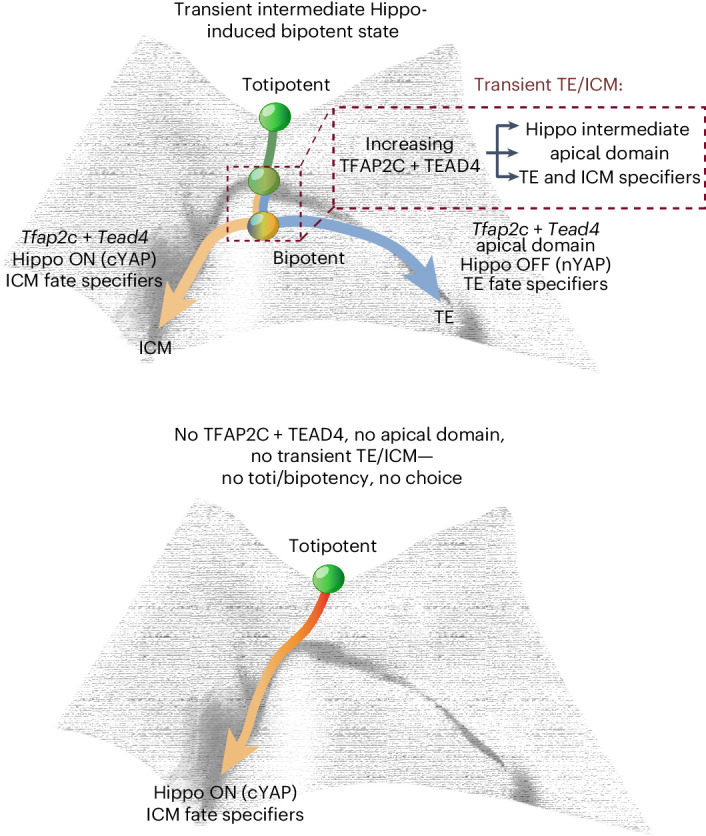
Model of the bipotential state mapped onto preimplantation development. In the bipotential state YAP is both nuclear (as in the TE) and cytoplasmic (as in the ICM). We propose here that zygotically expressed *Tead4* and *Tfap2c* accumulate in the nucleus and promote multilineage priming (expression of ICM- and TE-specific transcription factors and of Hippo components). Hippo signaling is intermediate (gray) (active AMOT, AMOTL2 and LATS2 in the cytoplasm), coincident with a preliminary apical domain. At the 16-cell stage, differential Hippo signaling mediated by *Tead4*, *Tfap2c* and *RhoA* and the TE-specific apical domain leads to diversification of the lineages: TE (blue) with Hippo OFF and ICM (beige) with Hippo ON. In the absence of *Tfap2c* and *Tead4*, no apical domain is formed and the transient TE/ICM composite state is abolished. As a result, the cells obtain an ICM-like state due to the low but phosphorylated cytoplasmic YAP, as well as the low-level expression of ICM fate specifiers. In the absence of the Hippo system, the cell fate specification is retarded and the cells are trapped in a null state without TE or ICM specification. We propose that totipotency diminishes to bipotency after zygotic genome activation initiates expression of *Tead4* and *Tfap2c*.

Our work shows that *Tfap2c* and *Tead4* establish a bipotent state and bistable switch in blastomeres before lineage diversification in the embryo (Fig. 7). These transcription factors regulate multiple parallel events relevant to the first lineage segregation: activation of TE and ICM genes, YAP subcellular localization, activation of Hippo signaling and aging of the apical domain. By promoting Hippo signaling on the one hand via activation of Hippo genes and inhibiting it on the other hand via the activation of polarity genes, TFAP2C and TEAD4 establish a switch that can support robust and immediate lineage diversification into TE and ICM, dependent on the asymmetric segregation of the apical domain.

YAP-induced negative feedback via Hippo signaling has been shown in other contexts to ensure that YAP-mediated regulation is transient and eventually extinguished^30^. In the context of the preimplantation embryo, we find that it is exploited to enable robust lineage diversification. Hippo signaling is inactivated due to compartmentalization of Hippo components by the apical domain in TE, which thus sustains YAP-induced TE specification. Notably, this negative feedback loop is often aberrantly inactivated in tumor cells^56^ that acquire hallmarks of the TE, such as a capacity for invasion. In contrast, the lack of an apical domain in the ICM enhances Hippo signaling, ultimately terminating the YAP-induced TE specifiers and allowing ICM fate (Fig. 7). Overexpression of TEAD4 or TFAP2C accelerate formation of the bipotent state, apical domain aging and lineage specification, whereas diminished levels of TEAD4 or TFAP2C have the opposite effect.

TE/ICM gene expression at the mid eight-cell stage, polarization at the late eight-cell stage and differential Hippo signaling at the early 16-cell stage, are closely spaced temporal events in mouse embryo development^20^. Our results suggest a molecular explanation that reconciles the relationship between the timing of these events. *Amot*/*Amotl2* and *Lats2* are expressed at the 8–16-cell stage^8,16^, and their expression before polarization leads to intermediate Hippo signaling. Apical domain formation subsequently couples polarization with the inactivation of Hippo signaling to establish the TE. Indeed, we found that earlier and elevated co-expression of TFAP2C or TEAD4, with active RHOA, accelerates this coupling^28^. We therefore suggest that activation of *Tfap2c, Tead4* and Rho GTPases initiates the polarity-dependent inhibition of the Hippo pathway. Polarized TE cells can display high levels of cytoplasmic, p-YAP, reminiscent of ICM cells. The apical domain does not always segregate completely during the asymmetric cell division to the 16-cell stage, leading to some cells carrying a small proportion of the apical domain. These mini-apical domains eventually disappear to make the cells apolar^7^. This imperfect apical domain segregation, together with the *Tfap2c*- or *Tead4*-induced bipotential, can lead to detectable cytoplasmic p-YAP in a subset of polarized blastomeres due to perdurance of p-YAP from the precursor, bipotential cell state. The bipotential state is eventually reconciled into distinct lineages via TTRhoA-mediated formation and aging of the apical domain in TE. Previously published datasets of TEAD4 Chip sequencing in trophoblast stem cells revealed direct binding sites of TEAD4 at *Amotl2*, *Lats2*, *Cdx2* and *Gata3* loci^57,58^. These data support our model and suggest that TFAP2C and TEAD4 establish the bipotent state by directly activating the expression of Hippo signaling components and TE/ICM lineage specifiers at the eight-cell stage.

We frequently detected p-YAP in the nucleus before lineage diversification. Interestingly, nuclear p-YAP was observed in sparsely plated mouse and human cells^33^. Mouse embryonic fibroblasts plated at low density displayed similar levels of p-YAP in the nucleus and cytoplasm; however, p-YAP was excluded from the nucleus when the actin cytoskeleton was depolymerized^33^. Thus, in the absence of cell–cell contact, F-actin cytoskeleton integrity promotes the nuclear localization of p-YAP, independent of contractility. Wada et al. also showed that phosphorylation of YAP is not sufficient to exclude YAP from nuclei and proposed that YAP subcellular localization is influenced by cell density^34^. It is possible that p-YAP can localize to and function in the nucleus in the two- to eight-cell stages before embryo compaction. It will be of interest to investigate the potential regulation of p-YAP subcellular localization in the embryo in future studies.

The expression of TE/ICM specifiers could be regulated by multiple mechanisms depending on the developmental stage^14,20,59^. Our results show that *Tfap2c* promotes expression of the TE regulators *Cdx2* and *Gata3*, the ICM regulators *Nanog* and *Oct4*, and the Hippo regulator *Amot* in bipotent cells. In addition, *Tead4* promotes the expression of *Cdx2* and *Gata3* and the Hippo regulators *Amotl2* and *Lats2* in bipotent cells. TFAP2C was recently shown to bind near and activate the Hippo activator *Amot*, the apical domain regulator *Pard6b*, the actin regulators *Cdc42ep1* and *Coro1*, the early TE genes *Tfeb* and *Itgb5* and the ICM genes *Nanog, Tdgf1* and *Nr5a2* at the eight-cell stage^12^. Upon TE commitment, TFAP2C was released from ICM genes and bound to late TE genes^12^. Intriguingly, the ICM regulator NR5A2, like TFAP2C, also served as a bipotency activator at the eight-cell stage^12^. Together, these findings suggest that a subset of opposing lineage specific regulators activate both lineages before commitment, coordinating a bistable switch that prepares bipotent blastomeres for a robust decision and differentiation into two fates. This bipotent state may ground the regulative property of the first lineage segregation, to allow the cells to tilt the balance of the cell fate easily and to self-adjust cell numbers of each lineage in response to the external environmental change^12,60^. This is particularly relevant for mammalian embryo development, as the first cell fate bifurcation is fulfilled without the involvement of external signaling^60^. We therefore reveal *Tfap2c* and *Tead4* as the candidate key upstream activators for such a regulative program.

We propose that the bistable switch established by *Tfap2c* and *Tead4* is a prerequisite for the first lineage segregation and that it facilitates the loss of totipotency. The zygote is unarguably functionally totipotent. However, we propose that the totipotency program is fully activated when both parental genomes share one nucleus for the first time, in each cell of the two-cell embryo, and the zygotic genome first awakens. In particular, zygotic *Tead4* and *Tfap2c* establish the bistable switch that can be reconciled by aging and asymmetric segregation of the apical domain, leading to robust lineage diversification.

## Methods

### Animals

This research has been carried out following regulations of the Animals (Scientific Procedures) Act 1986, Amendment Regulations 2012, reviewed by the University of Cambridge Animal Welfare and Ethical Review Body. Embryos were collected from 5–7-week-old F1 females (C57BI6xCBA) that had been super-ovulated by injection of 7.5 IU of pregnant mares’ serum gonadotropin followed by human chorionic gonadotropin (Intervet) 48 h later. F1 females were mated with F1 males. Two to four females were used for each experiment.

### Human embryos and ethical statements

Human embryos were donated from patients attending The Fertility Partnership (TFP) Oxford, with approval from the Human Fertilisation and Embryology Authority (Centre 0035, project RO198) and the Oxfordshire Research Ethics Committee (National Research Ethics Service (NRES) Committee South Central—Berkshire B; reference number 14/SC/0011). Experiments conducted in this work are compliant with International Society for Stem Cell Research guidelines. Informed consent was obtained from all patients. The study protocol and the manner in which it was conducted complied with all relevant regulations regarding the use of human study participants and was conducted in accordance with the criteria set by the Declaration of Helsinki. All new patients intending TFP Oxford for fertility treatment were given an information pack when they attended the evening meeting before starting treatment. An Information sheet about research projects using surplus eggs and embryos was included in the pack. Patients would not typically visit the clinic until several weeks after receiving this, giving time for them to consider whether or not they want to participate. All patients commencing their fertility treatment then arranged a routine new patient consultation appointment. At this visit doctors or nurses would check that the patient meets the inclusion criteria to participate in the study. This includes checking that the patient has, in a questionnaire supplied to all patients by the Human Fertilisation and Embryology Authority (form WT), agreed in principle to being approached about research projects involving their gametes (eggs). If so, they would ask the patient whether they wanted to participate in the study. A research nurse would always be available for further discussion of the projects if necessary. There was no patient compensation and embryos were not generated for research purposes.

### Mouse embryo culture and inhibitor treatments

Embryos were recovered at the zygote or two-cell stage in M2 medium and subsequently transferred to potassium simplex optimized medium (KSOM) for long-term culture, and staged for fixation as described previously^3^. C3-transferase was dissolved in distilled water and diluted in KSOM to 7 μg μl^−1^. For the control groups, the same dilutions of the vectors of different inhibitors were added to the medium.

### Microinjection

Microinjection was carried out as described previously^62^. In brief, embryos were placed in M2 medium on a glass slide with a depression and covered by a drop of mineral oil. Microinjection was performed with an Eppendorf Femtojet Microinjector. Negative capacitance was used to facilitate penetration through the membrane. Double-stranded (ds)RNA was injected at a concentration of 1 μg μl^−1^ (~1.547 pmol). Synthetic mRNAs were injected at the following concentration: EZRIN–RFP (500 ng μl^−1^; 278.8 fmol); *Tfap2c* (15 ng μl^−1^; 16.54 fmol); *Tead4* (15 ng μl^−1^; 21.01 fmol); RhoA-Q63L (3 ng μl^−1^; 6.705 fmol) and Cas9(100 ng μl^−1^; 55.76 fmol). All single-guide (sg)RNAs were injected at 25 ng μl^−1^ (13.94 pmol).

### Blastocyst reconstruction assay

The embryos at the late two-cell to early four-cell stage were injected with EZRIN–RFP only (control group) or with *Tfap2c* + *Tead4* + *RhoA-Q63L* mRNA with the concentration described in microinjection section, to better compare the signal between injected versus noninjected blastomeres. The embryos were then cultured until the 16-cell stage. The cells were disassociated at the 16-cell stage by placing the embryos in calcium- and magnesium-free M2 medium for 10 min followed by gentle glass pipetting. Polar and apolar cells were sorted manually by examining the presence of apical cap under a Sp5 confocal microscopy. Sixteen polar cells from each group were re-aggregated by gently blowing the cells into an indentation in a plastic dish containing KSOM covered by mineral oil. The re-aggregated embryos were then cultured until the late blastocyst stage 120 h post-hCG).

### Preparation of DNA constructs

pRN3P was used as the vector for all constructs as previously described^62^. pRN3P-Tfap2c, pRN3P-Tead4. Ezrin-Ruby, Ezrin-Venus, LifeAct-Ruby, GFP-Myl12b and RhoA-Q63L were as previously described^3^.

### mRNA, dsRNA and sgRNA preparation

For mRNA preparation, constructs for each mRNA were linearized using a restriction site downstream of the poly-A region. In vitro transcription was performed using the mMessage mMachine T3 kit (Thermo Fisher, AM1348), following the instructions from the manufacturer. mRNAs were then purified using the lithium chloride precipitation with ethanol wash method.

For sgRNA preparation, the sequences of sgRNAs were designed using CRISPR design tool website (http://crispr.mit.edu). The DNA fragment containing the T7 promoter, crispr (cr)RNA and sgRNA sequence were amplified using the Geneart gRNA kit (Thermo Fisher, A29377). sgRNAs were in vitro transcribed and purified using the gRNA Clean Up Kit (Thermo Fisher, A29377), following the manufacturer’s instructions.

All dsRNAs were designed using the E-RNAi website^63^ and were 350–500 bp in length. The specific targeting regions for each dsRNA were amplified from a mixture of mouse kidney, lung and liver complementary DNAs. The in vitro transcription reactions were performed using the MEGAscript T7 transcription kit (Thermo Fisher, AM1334) following the manufacturer’s instructions. dsRNAs were purified by lithium chloride precipitation.

### Immunofluorescence

Embryos were fixed in 4% paraformaldehyde in phosphate-buffered saline (PBS) for 20 min at room temperature, and then washed in PBST (0.1% Tween in PBS) three times. The embryos were permeabilized in 0.5% Triton X-100 in PBS for 20 min at room temperature, followed by PBST washing three times. The embryos were then transferred to blocking solution (3% bovine serum albumin in PBST) for 2 h and incubated with primary antibodies (diluted in blocking solution) at 4 °C overnight. After the incubation, embryos were washed in PBST three times, then incubated with secondary antibodies (1:500 in blocking solution) for 1 h at room temperature. Before mounting, the embryos were quickly stained (15 min) with 4,6-diamidino-2-phenylindole (DAPI) (1:1,000 dilution, in PBST, Life Technologies, D3571), followed by two washes in PBST. The primary antibodies were as follows: rabbit polyclonal anti-Pard6b (Santa Cruz, sc-67393, 1:200), mouse monoclonal anti-GFP (Nacalai Tesque Inc., 04404-84, 1:500), mouse monoclonal anti-Tfap2c (Santa Cruz, sc-12762, 1:200), goat monoclonal anti-Tfap2c (R&D Systems, AF5059-SP, 1:200), rabbit monoclonal anti-Tead4 (Abcam, ab97460, 1:200), mouse monoclonal anti-Tead4 (Abcam, ab58310, 1:100), goat monoclonal anti-Sox17 (R&D Systems, af1924), mouse monoclonal anti Cdx2 (Biogenex, MU392-UC, 1:200), rabbit monoclonal anti-Nanog (Abcam, ab80892, 1:200), rabbit monoclonal anti-phosphorylated-Yap (Cell Signaling Technologies, 4911S, 1:200), mouse monoclonal anti-Yap (Santa Cruz, sc-101199, 1:200), goat polyclonal anti-Amot (Santa Cruz, sc-82491, 1:1,000), and rabbit polyclonal anti-Klf5 (Proteintech, 21017–1-AP). The secondary antibodies were Alexa Fluor 568 Donkey anti-gGoat (A-11057, Thermo Fisher Scientific), Alexa Fluor 488 Donkey anti-Mouse, (A-21202, Thermo Fisher Scientific), Alexa Fluor 568 Donkey anti-Mouse (A10037, Thermo Fisher Scientific), Alexa Fluor 647 Donkey anti-Mouse (A31571, Thermo Fisher Scientific), Alexa Fluor 568 Donkey anti-Rabbit (A10042, Thermo Fisher Scientific) and Alexa Fluor 647 Donkey anti-Rabbit (A-31573, Thermo Fisher Scientific).

### Imaging and data processing

Imaging was carried out on a Leica-SP5 or a Leica-SP8 confocal using a Leica 1.4 NA 63× oil (Harmonic Compound (HC) Plan (PL) Apochromatic (APO)) objective. Images were processed with Fiji software^55,64^. For the analysis of nucleo-cytoplasmic signal intensity ratio, the region of the nucleus and a cytoplasmic region of the same size were cropped and the mean signal extracted using the Fiji region of interest (ROI) function. To normalize signals to the level of DAPI fluorescence, the Fiji ROI function was used to extract the nuclear region stained to reveal specific proteins and for the equivalent DAPI channel and normalized using the formula: protein of interest / DAPI. For apical enrichment or membrane localization analysis, a freehand line of the width of 0.5 μm was drawn along the cell–contact free surface (apical domain), cell–contact (basal) or cytoplasmic area of the cell, signal intensity was obtained via the ROI function of Fiji. The apical/basal or membrane/cytoplasmic signal intensity ratio is calculated as: region 1 / region 2. Cells on the same plane were subjected to this analysis. The eight-cell embryos were staged by the level of compaction and the hours of postdivision of the last cell at the four-cell stage. Compaction was assessed by measuring the intercellular blastomere angle in the mid-plane between adjacent cells (as described previously^3^) by using the Fiji angle function.

For live-imaging, time-lapse recordings of embryos were carried out using a spinning disk or a Leica-SP5 scanning confocal. Time-lapse frames were acquired every 20–30 min. Images were acquired using a 3–4 μm *Z*-step. Images were processed with Fiji software. Correlations were calculated using Prism software (http://www.graphpad.com).

### Statistics

Statistical methods are indicated for every experiment in the corresponding figure legends. Qualitative data are presented as a contingency table and were analyzed using Fisher’s exact test. The normality of quantitative data was first analyzed using D’Agostino’s *K*^2^. If data showed a normal distribution, then for comparison of two or multiple samples, an unpaired two-tailed Student’s *t*-test (two experimental groups) or a one-way analysis of variance (ANOVA) test (more than two experimental groups) was used to analyze statistical significance. For data that did not present a normal distribution, a two-sided Mann–Whitney *U*-test (two experimental groups) or a Kruskal–Wallis test with a Dunn’s multiple comparison test (more than two experimental groups) was used to test statistical significance. To determine the influence of different groups in multiple variants, two-way ANOVA was performed. Statistical analyses were performed using Prism software (http://www.graphpad.com).

### Human ATAC-sequencing data analyses and binding sites prediction

Published ATAC-sequencing data for both mouse and human preimplantation embryos were retrieved from the Gene Expression Omnibus (accession number: GSE101571)^55^ and were aligned to mm9 or hg19 reference genome by Bowtie (version 2.2.2). After removing all PCR duplicates, unmapped and non-uniquely mapped reads, the processed mapped reads were normalized by the numbers of reads per kilobase of bin per million of read sequenced and further *Z*-score transformation. The processed ATAC-sequencing tracks were visualized in the University of California, Santa Cruz genome browser. The motif sequences of TFAP2C and TEAD4 obtained from HOMER motif database were aligned to the promoter and enhancer open chromatin regions of a selection of TE/ICM and HIPPO pathway markers to predict the dynamic of accessible TFAP2C and TEAD4 binding sites across various developmental stages of mouse and human embryos, encompassing two-cell, four-cell, eight-cell and ICM stages.

### Reporting summary

Further information on research design is available in the Nature Portfolio Reporting Summary linked to this article.

## Online content

Any methods, additional references, Nature Portfolio reporting summaries, source data, extended data, supplementary information, acknowledgements, peer review information; details of author contributions and competing interests; and statements of data and code availability are available at 10.1038/s41594-024-01311-9.

### Supplementary information

Supplementary Information Source images for Figs. 1–6 and source images for Extended Data Figs. 1–6.

Reporting Summary

### Source data

Source data Fig. 1 Quantifications for Fig. 1.

Source data Fig. 2 Quantifications for Fig. 2.

Source data Fig. 3 Quantifications for Fig. 3.

Source data Fig. 4 Quantifications for Fig. 4.

Source data Fig. 5 Quantifications for Fig. 5.

Source data Fig. 6 Quantifications for Fig. 6.

Source data Extended Data Fig. 1/Table 1 Quantifications for Extended Data Fig. 1.

Source data Extended Data Fig. 4/Table 4 Quantifications for Extended Data Fig. 4.

Source data Extended Data Fig. 6/Table 6 Quantifications for Extended Data Fig. 6.

## Data Availability

The bulk RNA-sequencing data of *Tfap2c* and *Tead4* RNAi at the eight-cell stage mouse embryo were deposited as previously described^28^ (GSE124755). All other raw data for making the graphs in the paper, as well as the raw images used in figures can be found in the Source data and Supplementary information sections in the manuscript. Source data are provided with this paper.
