# Supplementary material for: Tead4 and Tfap2c generate bipotency and a bistable switch in totipotent embryos to promote robust lineage diversification
Source: Nat Struct Mol Biol. 2024 May 24;31(6):964–76. doi: 10.1038/s41594-024-01311-9 (PMC11189297; doi:10.1038/s41594-024-01311-9)

nature structural & molecular biology

Supplementary information https://doi.org/10.1038/s41594-024-01311-9

Tead4 and *Tfap2c* generate bipotency and a bistable switch in totipotent embryos to promote robust lineage diversification

In the format provided by the authors and unedited

Supplementary Fig.1

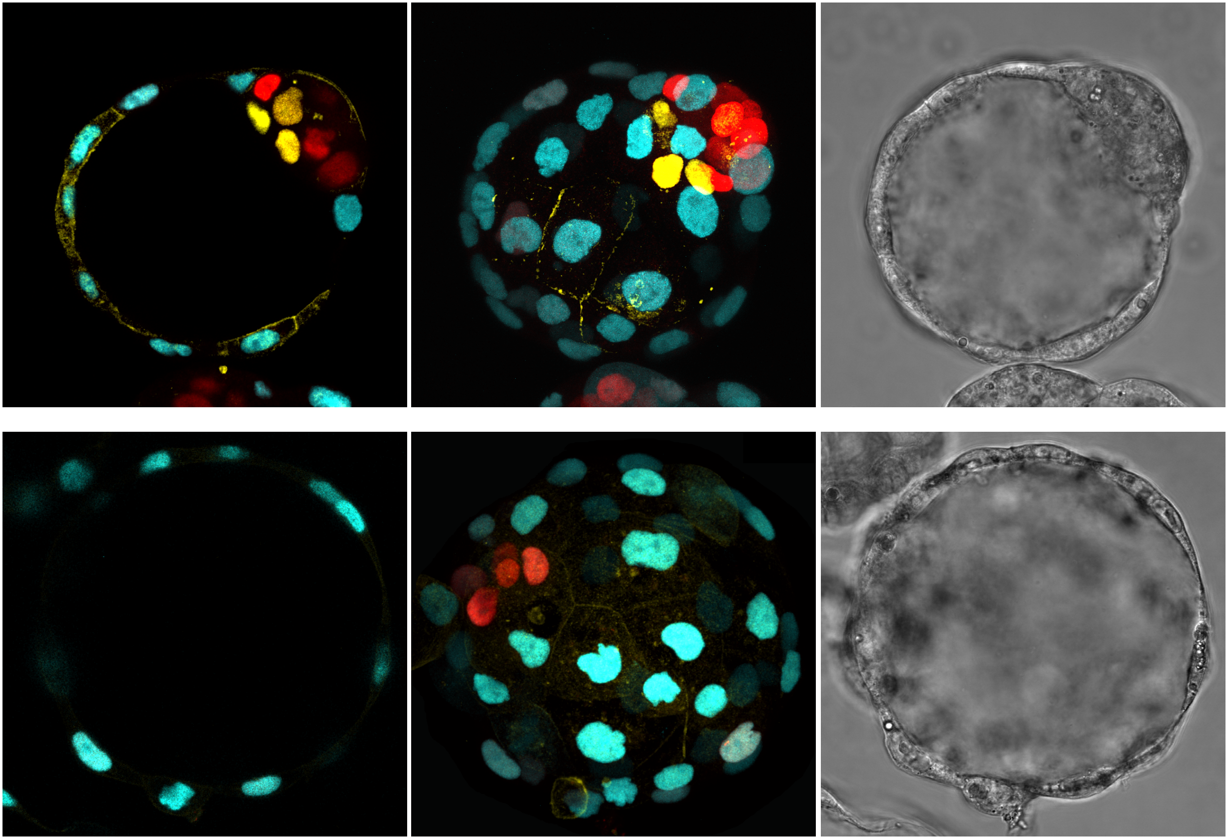

Supplementary Fig.2

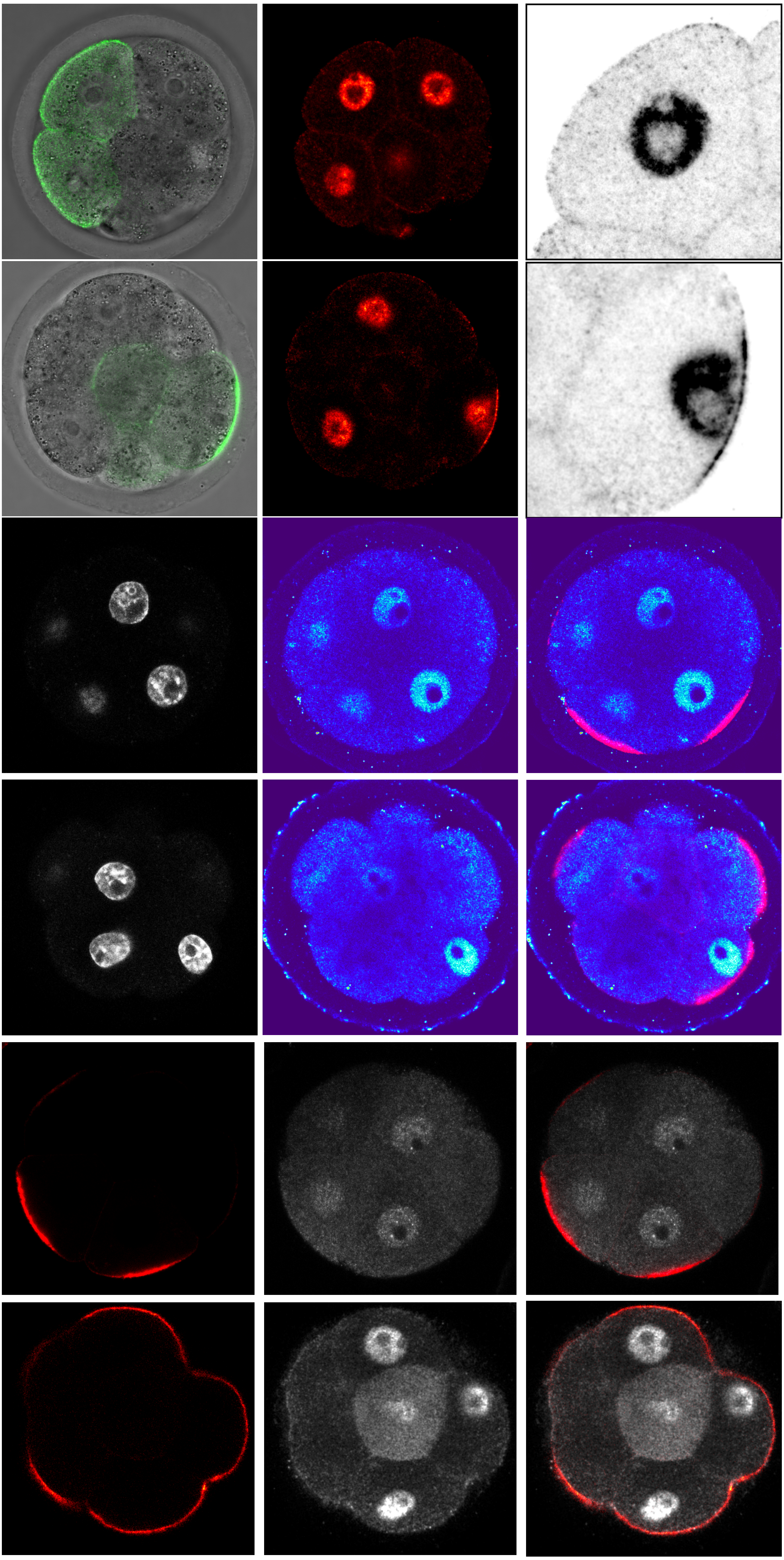

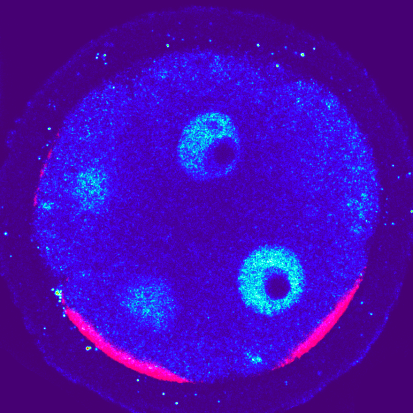

Supplementary Fig.3

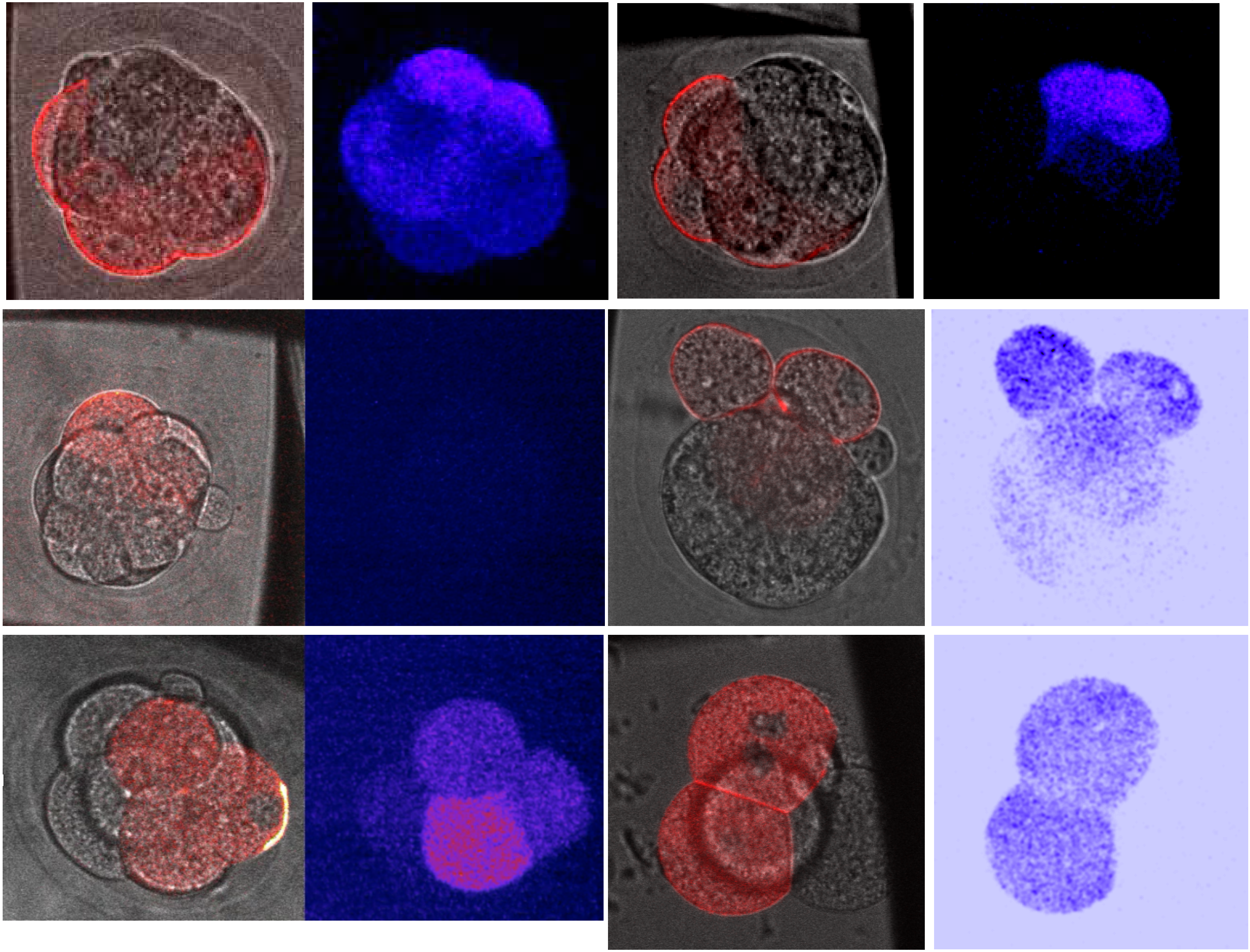

Supplementary Fig.4

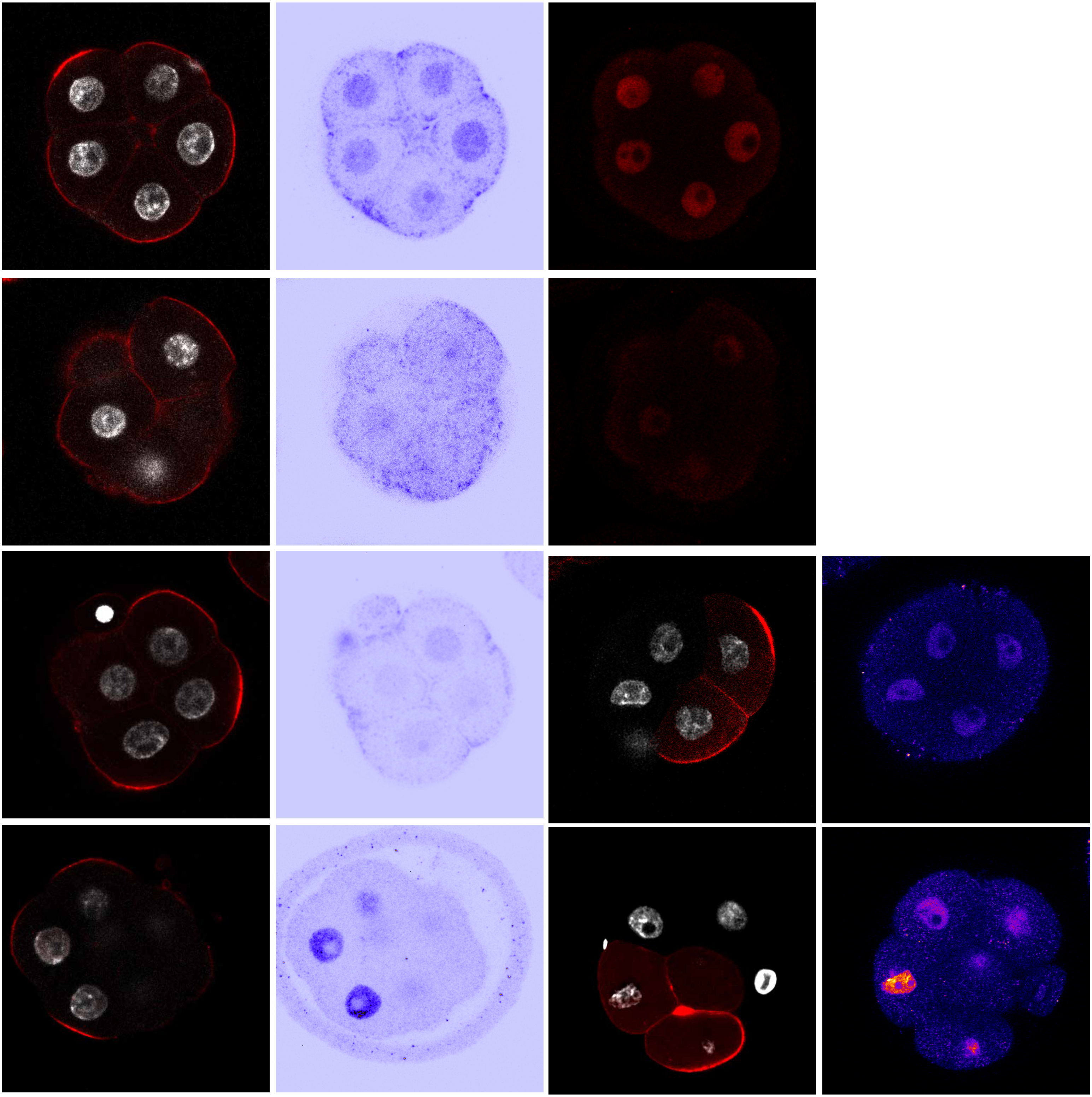

Supplementary Fig.5

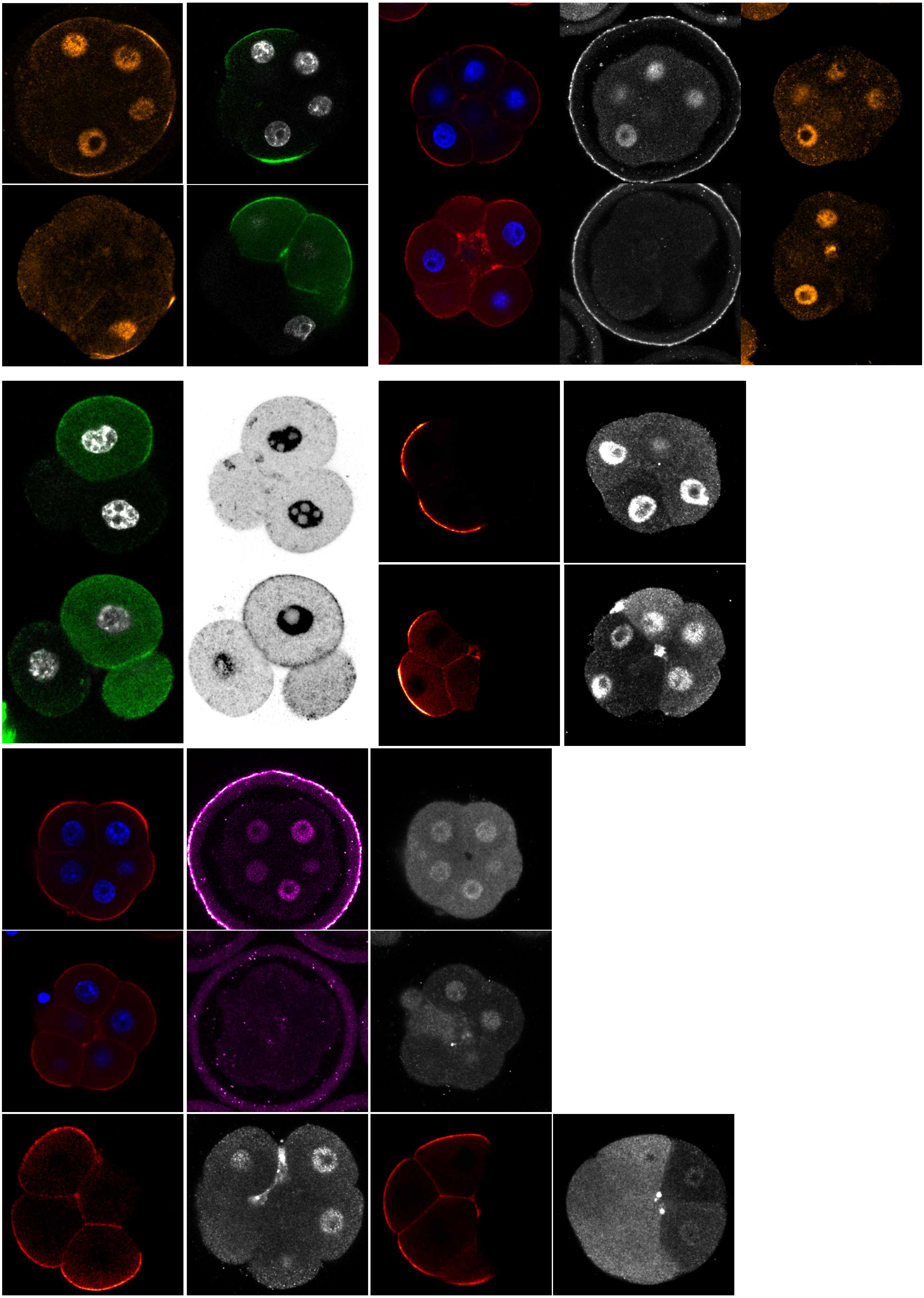

Supplementary Fig. 6

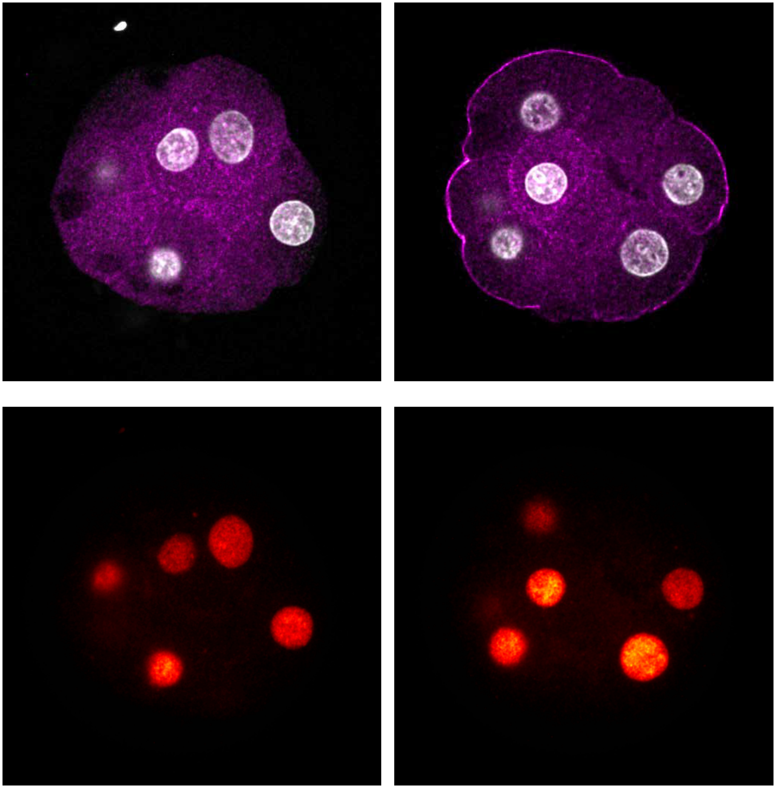

Supplementary Fig.7

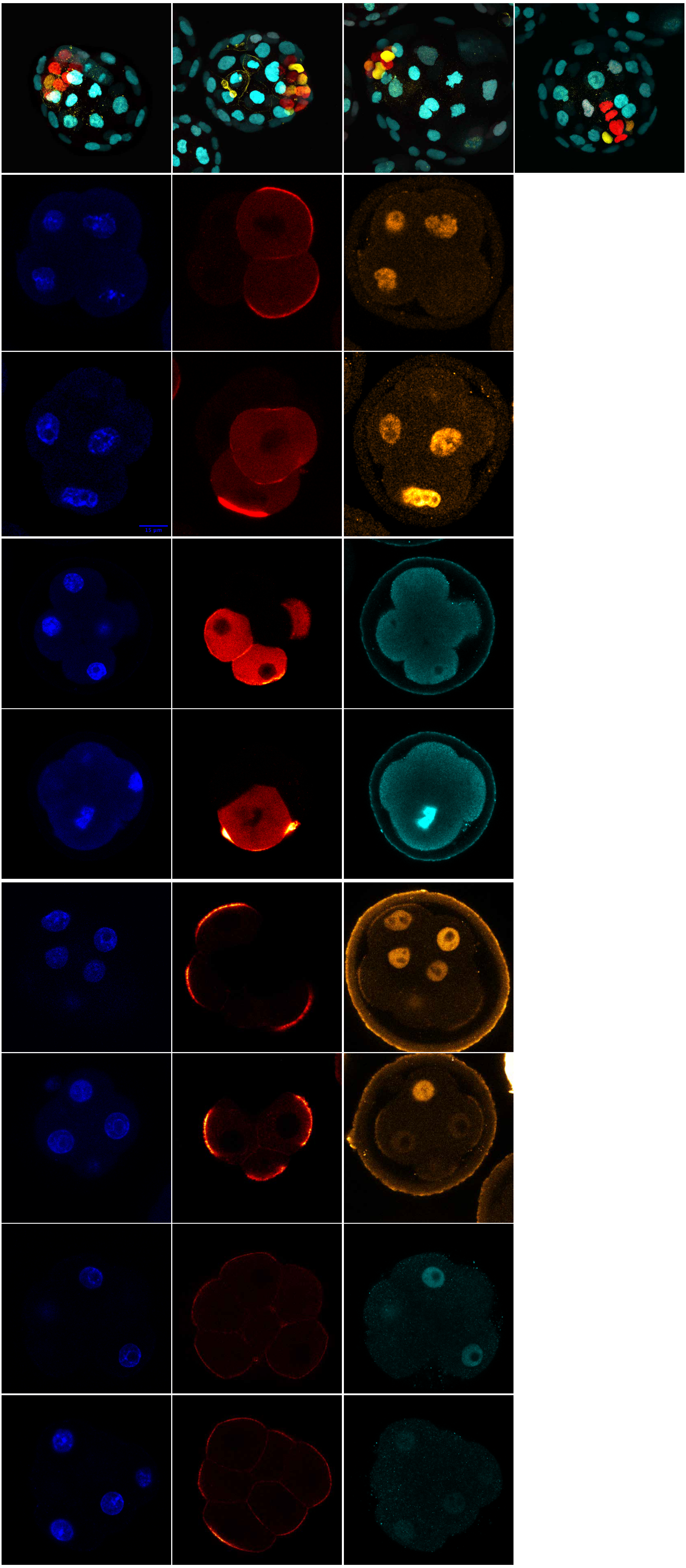

Supplementary Fig.8

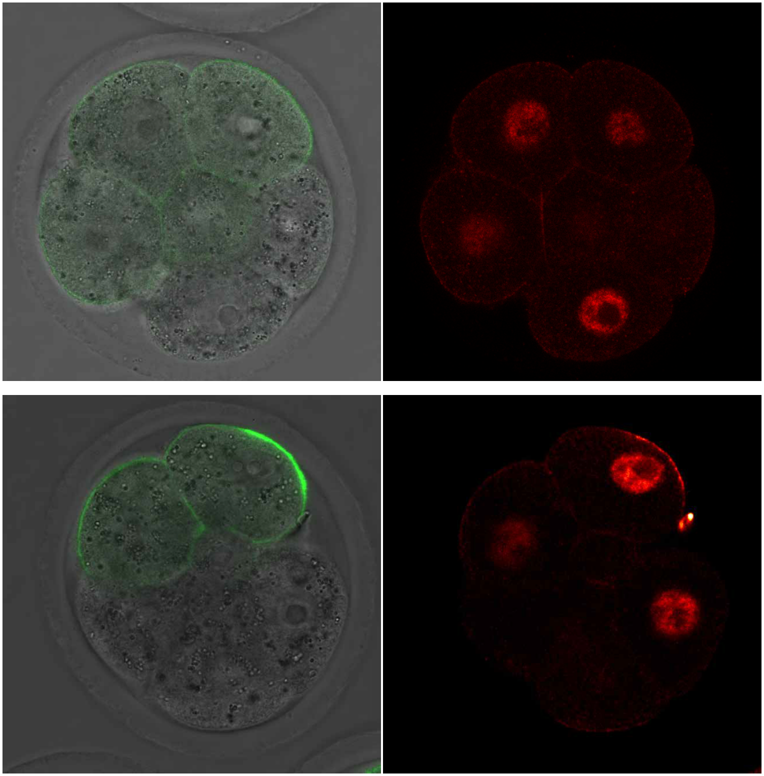

Supplementary Fig.9

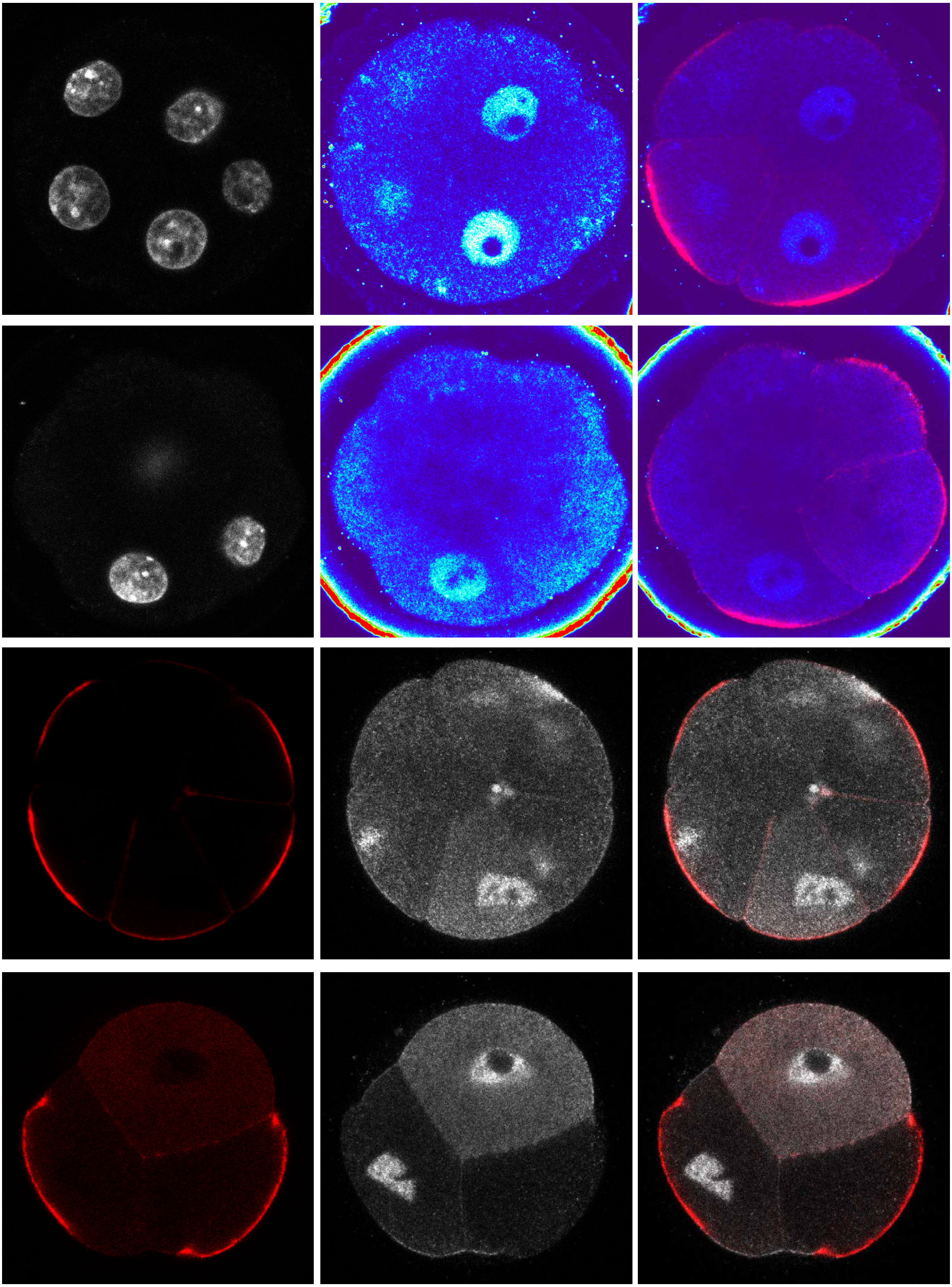

Supplementary Fig.10

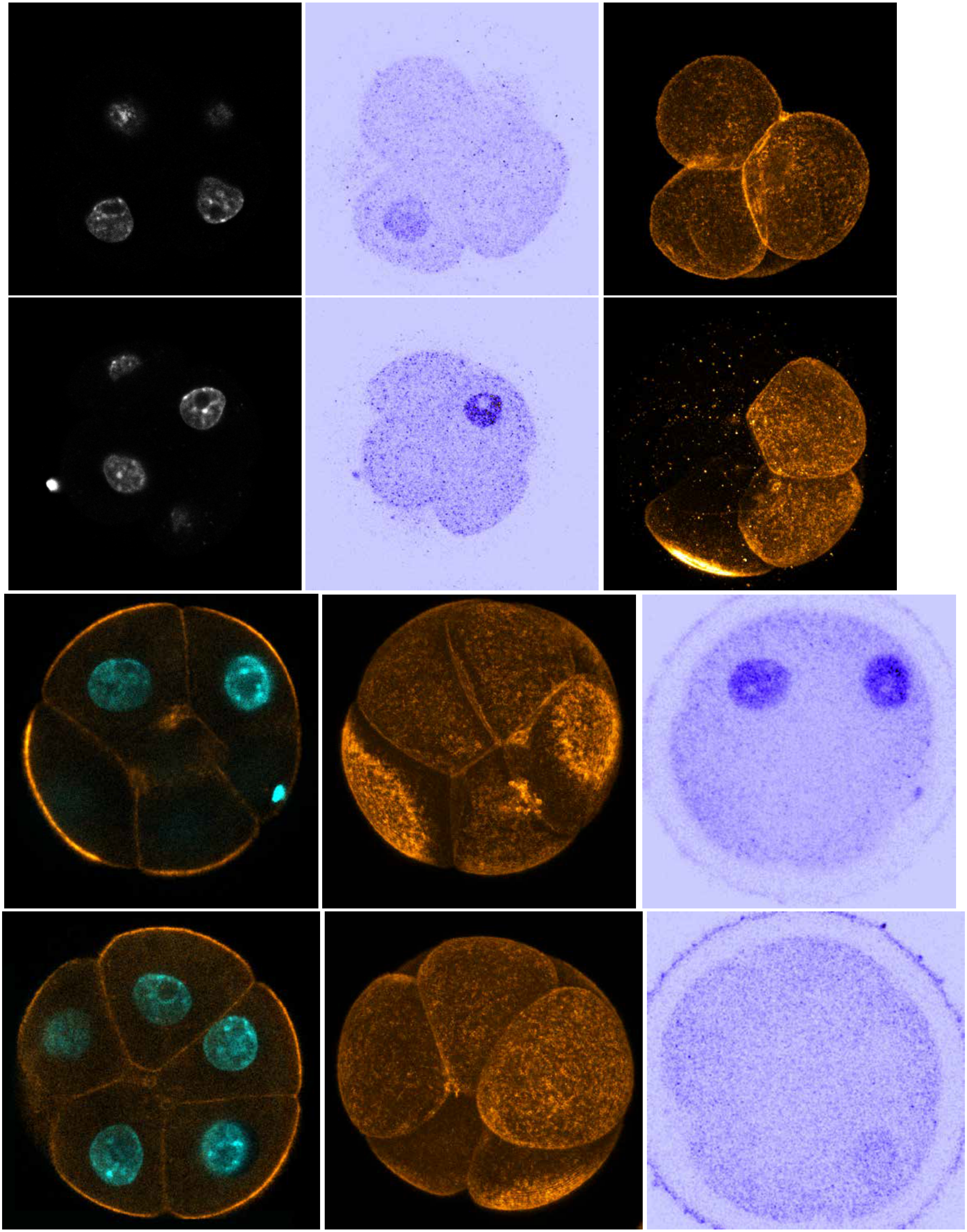

Supplementary Fig.11

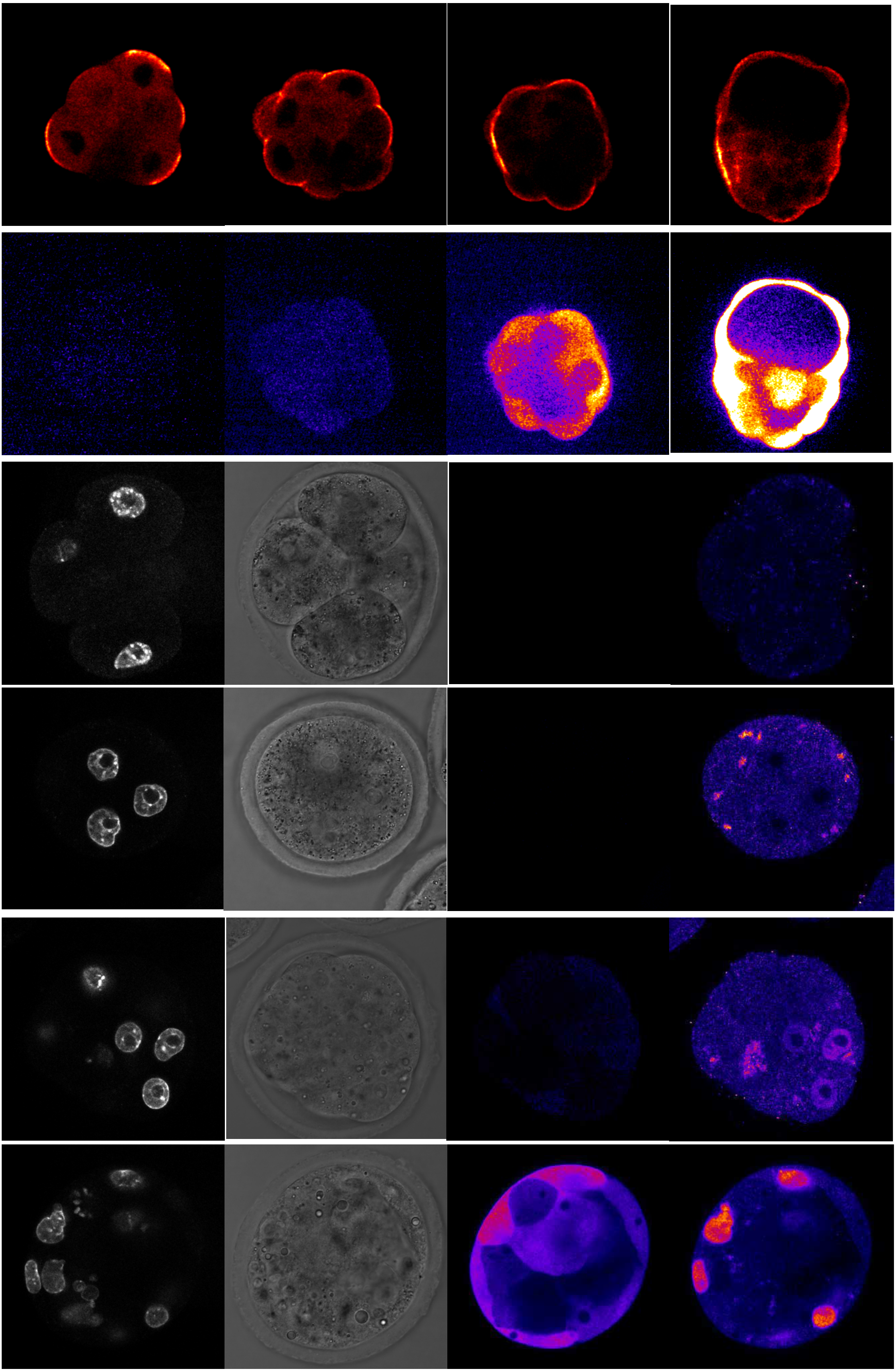

Supplementary Fig.12

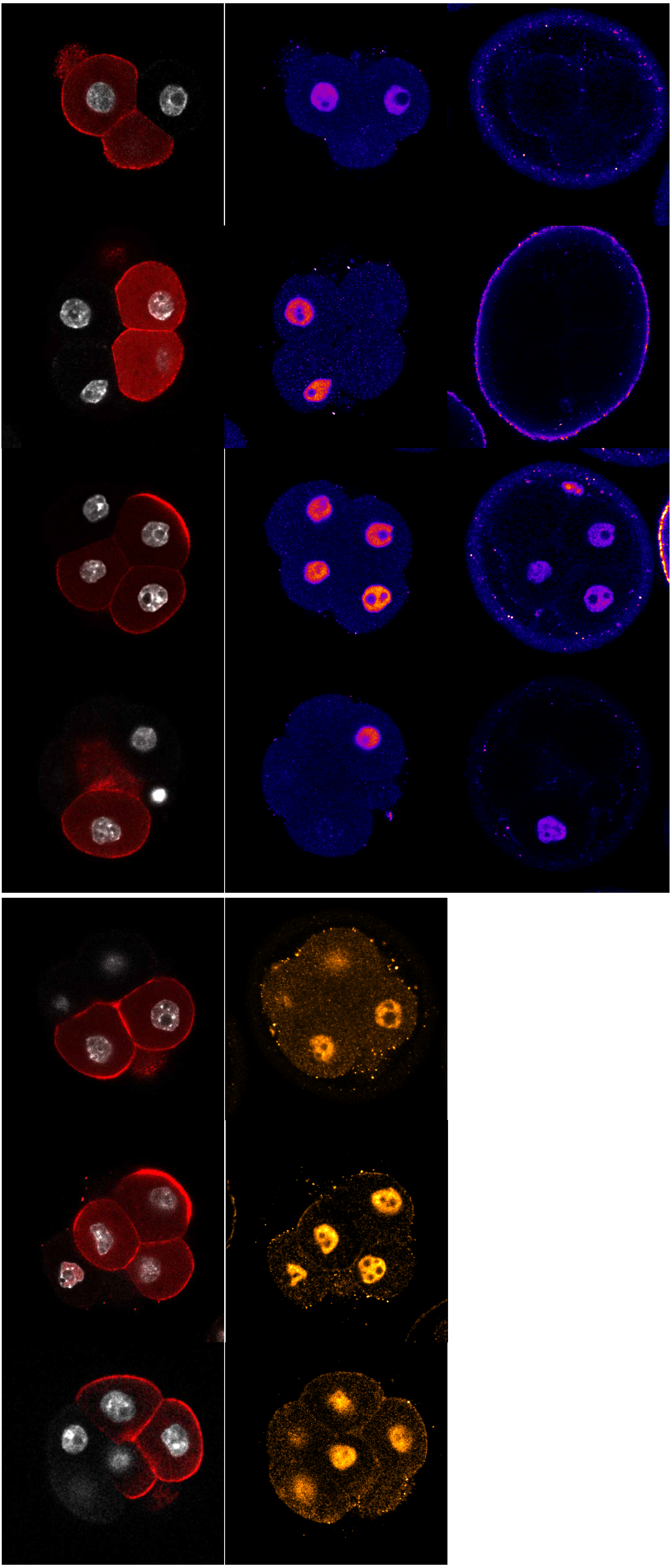

Supplement: Supplementary file 1 — Source images for Figs. 1–6 and source images for Extended Data Figs. 1–6. [file 41594_2024_1311_MOESM1_ESM.pdf]
