# Supplementary material for: Tead4 and Tfap2c generate bipotency and a bistable switch in totipotent embryos to promote robust lineage diversification
Source: Nat Struct Mol Biol. 2024 May 24;31(6):964–76. doi: 10.1038/s41594-024-01311-9 (PMC11189297; doi:10.1038/s41594-024-01311-9)
Supplement: Supplementary file 2 — Reporting Summary [file 41594_2024_1311_MOESM2_ESM.pdf]

nature portfolio

Corresponding author(s): Magdalena Zernicka-Goetz

Last updated by author(s): Mar 16, 2024

Reporting Summary

Nature Portfolio wishes to improve the reproducibility of the work that we publish. This form provides structure for consistency and transparency in reporting. For further information on Nature Portfolio policies, see our Editorial Policies and the Editorial Policy Checklist.

Statistics

For all statistical analyses, confirm that the following items are present in the figure legend, table legend, main text, or Methods section.

n/a	Confirmed	
☐	☒	The exact sample size (*n*) for each experimental group/condition, given as a discrete number and unit of measurement
☐	☒	A statement on whether measurements were taken from distinct samples or whether the same sample was measured repeatedly
☐	☒	The statistical test(s) used AND whether they are one- or two-sided<br>*Only common tests should be described solely by name; describe more complex techniques in the Methods section.*
☐	☒	A description of all covariates tested
☐	☒	A description of any assumptions or corrections, such as tests of normality and adjustment for multiple comparisons
☐	☒	A full description of the statistical parameters including central tendency (e.g. means) or other basic estimates (e.g. regression coefficient) AND variation (e.g. standard deviation) or associated estimates of uncertainty (e.g. confidence intervals)
☐	☒	For null hypothesis testing, the test statistic (e.g. *F*, *t*, *r*) with confidence intervals, effect sizes, degrees of freedom and *P* value noted<br>*Give P values as exact values whenever suitable.*
☐	☒	For Bayesian analysis, information on the choice of priors and Markov chain Monte Carlo settings
☐	☒	For hierarchical and complex designs, identification of the appropriate level for tests and full reporting of outcomes
☒	☐	Estimates of effect sizes (e.g. Cohen's *d*, Pearson's *r*), indicating how they were calculated

Our web collection on statistics for biologists contains articles on many of the points above.

Software and code

Policy information about availability of computer code

Data collection	All images are collected through Confocal mircoscopy (Leica Sp5 or Sp8), or Spinning disk confocal microscopy (Nikon). None of the custom code applies.
Data analysis	Any measurements on images are analysed through ImageJ software. The quantifications are obtained through plugins on ImageJ software (Fiji v2.14.0) as indicated in "material and method" section. The statistical data are obtained through prism software (Prism v8.2.0).

For manuscripts utilizing custom algorithms or software that are central to the research but not yet described in published literature, software must be made available to editors and reviewers. We strongly encourage code deposition in a community repository (e.g. GitHub). See the Nature Portfolio guidelines for submitting code & software for further information.

Data

Policy information about availability of data

All manuscripts must include a data availability statement. This statement should provide the following information, where applicable:

- Accession codes, unique identifiers, or web links for publicly available datasets
- A description of any restrictions on data availability
- For clinical datasets or third party data, please ensure that the statement adheres to our policy

The Bulk RNA-sequencing data of Tfap2c and Tead4 RNAi at the 8-cell stage mouse embryo was deposited as previously described28 (GSE124755). All other raw data for making the graphs in the paper, as well as the raw images used in figures can be found in the "Source Data files" section in the manuscript.

Research involving human participants, their data, or biological material

Policy information about studies with human participants or human data. See also policy information about sex, gender (identity/presentation), and sexual orientation and race, ethnicity and racism.

Reporting on sex and gender	No sex or gender related studies involved.
Reporting on race, ethnicity, or other socially relevant groupings	These information are not allowed to obtain under the current ethical provision
Population characteristics	No population studies involved
Recruitment	Informed consent was obtained from all patients. The study protocol and the manner in which it was conducted complied with all relevant regulations regarding the use of human study participants and was conducted in accordance with the criteria set by the Declaration of Helsinki. All new patients intending TFP Oxford for fertility treatment were given an information pack when they attended the evening meeting before starting treatment. An Information sheet about research projects using surplus eggs and embryos was included in the pack. Patients would not typically visit the clinic until several weeks after receiving this, giving time for them to consider whether or not they want to participate. All patients commencing their fertility treatment then arranged a routine new patient consultation appointment. At this visit doctors/nurses would check that the patient meets the inclusion criteria to participate in the study. This includes checking that the patient has, in a questionnaire supplied to ALL patients by the HFEA (Form WT), agreed in principle to being approached about research projects involving their gametes (eggs). If so, they would ask the patient if they wanted to participate in the study. A research nurse would always be available for further discussion of the projects if necessary. There was no patient compensation and embryos were not generated for research purposes.
Ethics oversight	Human embryos were donated from patients attending The Fertility Partnership (TFP) Oxford with approval from the Human Fertilisation and Embryology Authority (Centre 0035, project RO198) and the Oxfordshire Research Ethics Committee (NRES Committee South Central—Berkshire B; Reference number 14/SC/0011). Experiments conducted in this work is compliance to ISSCR guidelines.

Note that full information on the approval of the study protocol must also be provided in the manuscript.

Field-specific reporting

Please select the one below that is the best fit for your research. If you are not sure, read the appropriate sections before making your selection.

☒ Life sciences ☐ Behavioural & social sciences ☐ Ecological, evolutionary & environmental sciences

For a reference copy of the document with all sections, see nature.com/documents/nr-reporting-summary-flat.pdf

Life sciences study design

All studies must disclose on these points even when the disclosure is negative.

Sample size	No sample size calculation was performed. The sample size was not predetermined.
Data exclusions	The unhealthily developed embryos (judged by mal-morphologies, such as the fragmentation of the embryos or majority of the cell death, or developmental retardation) have been excluded from the analysis.
Replication	For each experiment at least two biological replicates have been obtained. All biological replicates were successful and consistent in the trend with the final conclusion.
Randomization	The embryos are randomly assigned for treatments or control groups.
Blinding	The group allocation is not blinded as no other methods can be used to identify the treatment groups.

Reporting for specific materials, systems and methods

We require information from authors about some types of materials, experimental systems and methods used in many studies. Here, indicate whether each material, system or method listed is relevant to your study. If you are not sure if a list item applies to your research, read the appropriate section before selecting a response.

Materials & experimental systems

n/a	Involved in the study	
☐	☒	Antibodies
☒	☐	Eukaryotic cell lines
☒	☐	Palaeontology and archaeology
☐	☒	Animals and other organisms
☒	☐	Clinical data
☒	☐	Dual use research of concern
☒	☐	Plants

Methods

n/a	Involved in the study	
☒	☐	ChIP-seq
☒	☐	Flow cytometry
☒	☐	MRI-based neuroimaging

Antibodies

Antibodies used

Primary antibodies: rabbit polyclonal anti-Pard6b (Santa Cruz, sc-67393, 1:200); mouse monoclonal anti-GFP (Nacalai Tesque Inc., 04404-84, 1:500). mouse monoclonal anti-Tfap2c (Santa Cruz, sc-12762, (6E4/4), 1:200); goat monoclonal anti-Tfap2c (R&D Systems, AF5059-SP, 1:200); rabbit monoclonal anti-Tead4 (Abcam, ab97460, 1:200); mouse monoclonal anti-Tead4 (Abcam, ab58310, 1:100); goatmonoclonal anti Sox17 (R&D Systems, af1924); mouse monoclonal anti-Cdx2 (Launch Diagnostics, MU392-UC (Biogenex), 1:200);rabbit monoclonal anti Nanog (Abcam, ab80892, 1:200); mouse monoclonal anti-Tjp1 (Thermo Fisher Scientific, 33-9100, 1:200);rabbit monoclonal anti-phosphorylated-Yap (Cell Signaling Technologies, 4911S, 1:200); mouse monoclonal anti-Yap (Santa Cruz,sc-101199, 1:200); rabbit monoclonal anti-di-phosphorylated MRLC (Cell Signaling Technologies, 3674P, 1:100); goat polyclonal anti-Amot (Santa Cruz, sc-82491, 1:1000); rabbit polyclonal anti-Klf5 (Proteintech, 21017–1-AP). Secondary antibodies: Alexa Fluor 568 Donkey anti-Goat (A-11057, ThermoFisher Scientific);Alexa Fluor 488 Donkey anti-Mouse, (A-21202, ThermoFisher Scientific); Alexa Fluor 568 Donkey anti-Mouse (A10037, ThermoFisherScientific); Alexa Fluor 647 Donkey anti-Mouse (A31571, ThermoFisher Scientific); Alexa Fluor 568 Donkey anti-Rabbit (A10042,ThermoFisher Scientific); Alexa Fluor 647 Donkey anti-Rabbit (A-31573, ThermoFisher Scientific).

Validation

All antibodies are validated either by knockdown or overexpression experiments in the mouse embryo. Cdx2, Nanog, Oct4 and Gata3 antibodies are validated by lineage localisation.

Animals and other research organisms

Policy information about studies involving animals; ARRIVE guidelines recommended for reporting animal research, and Sex and Gender in Research

Laboratory animals

Embryos were collected from F1 females (C57Bl6xCBA) that had been super-ovulated by injection of 7.5 IU of pregnant mares' serum gonadotropin followed by human chorionic gonadotropin (Intervet) 48 h later. F1 females were mated with F1 males. The mice are maintained under 12 light/12 dark cycle, the temperature of 18-23°C and with 40-60% humidity .

Wild animals

No wild animals used

Reporting on sex

No sex studies invilved

Field-collected samples

No field collected samples

Ethics oversight

This research has been carried out following regulations of the Animals (Scientific Procedures) Act 1986 - Amendment Regulations 2012 - reviewed by the University of Cambridge Animal Welfare and Ethical Review Body.

Note that full information on the approval of the study protocol must also be provided in the manuscript.
